# Semisynthetic and SAR Studies of Amide Derivatives of Neocrotocembraneic Acid as Potential Antitumor Agents

**DOI:** 10.3390/molecules21111581

**Published:** 2016-11-19

**Authors:** Hai Shang, Ling-Yu Li, Wei-Hua Cheng, Jun Luo, Hong-Wu Zhang, Zhong-Mei Zou

**Affiliations:** 1 Institute of Medicinal Plant Development, Chinese Academy of Medical Sciences and Peking Union Medical College, Beijing 100193, China; shanghai0503@163.com (H.S.); bluehill07@163.com (L.-Y.L.); cheng1083@163.com (W.-H.C.); luojun1371898@163.com (J.L.); hwzhang@implad.ac.cn (H.-W.Z); 2 School of Life Science and Engineering, Southwest University of Science and Technology, Mianyang 621010, China

**Keywords:** cembranoid type diterpene, neocrotocembraneic acid, structural modification, antitumor activity, anti-multidrug resistance

## Abstract

A series of novel amide derivatives of cembranoid neocrotocembraneic acid were designed and synthesized. The antiproliferative activities of these derivatives were evaluated against three human tumor cell lines (the human cervical cancer cell line HeLa, chronic myeloid leukemia cell line K562 and leukemia multidrug-resistant cell line K562/A02). Some of the synthesized compounds exhibited moderate to good activity against all three cancer cell lines. Particularly, compound **8a** exhibited more potent antiproliferative activity than the reference drug etoposide against drug-resistant cell line K562/A02, indicating that it possessed a great potential for further development as a multidrug resistance modulator by structural modification.

## 1. Introduction

Cembranoids are natural diterpenes possessing 14-membered macrocyclic rings substituted by an isopropyl residue at C-1 and by three symmetrically disposed methyl groups at positions C-4, C-8, and C-12 [1]. They have been encountered among diverse terrestrial plants, insects and marine sources [2,3]. Up until now, more than 300 natural cembranoid derivatives were isolated and their structural diversity is mainly reflected in the different degrees of oxidation of the macrocyclic framework [4]. Cembranoids also exhibit a broad-ranging bioactivity profile that includes antitumor [5,6,7,8,9,10,11,12], anti-inflammatory [13,14,15,16,17], antimicrobial [18,19], immunomodulatory [20], and osteoporosis-preventive activity [21]. It is noteworthy that the antitumor activity is the most remarkable property of this class of diterpenoids.

**Figure 1:**
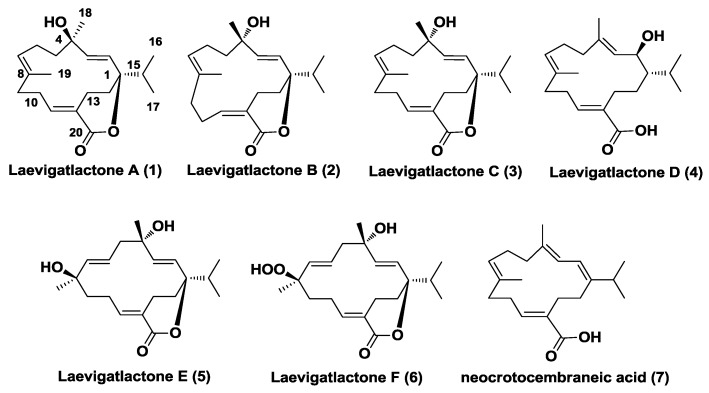
Cembrane diterpenoids were isolated from the leaves of *C. laevigatus* Vahl.

*Croton laevigatus* Vahl. (Euphorbiaceae) is an arbor that is found mainly in the Yunnan, Guangdong, and Hainan provinces of China. Its roots and leaves have been commonly used as a folk medicine in the Dai nationality of China for the treatment of injuries from falls and fractures, malaria and stomachaches [22]. In our previous work, we have reported studies on the chemical composition of the leaves of *C. laevigatus* Vahl. and seven cembranoids were isolated (Figure 1) [23]. The antitumor activity of these compounds was evaluated against the human cervical cancer cell line HeLa and neocrotocembraneic acid showed modest cytotoxic activity (IC_50_ = 45.4 μM). Moreover, this natural product has a high content in *C. laevigatus* Vahl. (0.7% of the plant’s dry weight) which laid the material foundation for the structural modification. However, there was no study on the structural modification and the structure-activity relationship (SAR) of neocrotocembraneic acid related to the antitumor purpose. This stimulated us with great interest to focus on the structural modification of neocrotocembraneic acid in order to obtain the initial structure-activity relationship and find novel derivatives with potential antitumor activity. According to the characteristics of this molecular structure, we believed that the carboxyl group was very suitable for modifications to obtain structurally diverse analogues in the initial study. A generally useful modification of carboxylic acid is the introduction of substituted amines by the amidation reaction, which may serve as new hydrogen bond donors and receptors to increase binding affinity to the enzyme and also to improve the physicochemical properties. Thus, in this study, a series of amide derivatives of neocrotocembraneic acid were designed and synthesized. In order to further investigate of the effect of *N*-substituents on antitumor activity, different substituted amines (heterocyclic amine, benzylamine, phenethylamine and aliphatic amines) were introduced. The antiproliferative activities of the synthesized compounds against the human cervical cancer cell line (HeLa), chronic myeloid leukemia cell line (K562) and leukemia multidrug-resistant cell line (K562/A02) were evaluated and a preliminary SAR study of these compounds was discussed.

## 2. Results and Discussion

### 2.1. Chemistry

**Scheme 1:**
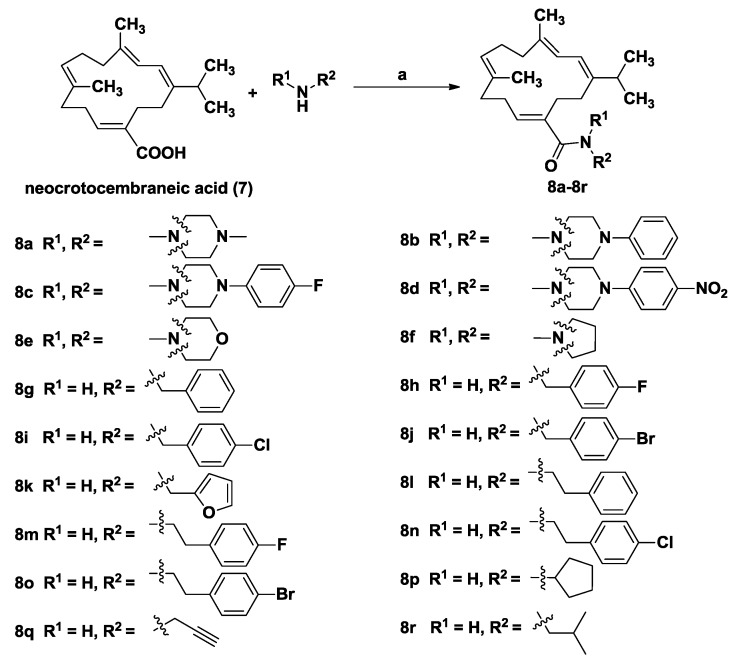
Synthesis of the amide derivatives **8a**–**8r**. *Reagents and conditions*: (**a**) HOBt, EDCI, CH_2_Cl_2_.

The synthesis of derivatives **8a**–**8r** is outlined in Scheme 1. Amidation of neocrotocembraneic acid with commercially available amines using 1-(3-dimethylaminopropyl)-3-ethylcarbodiimide hydrochloride (EDCI) and 1-Hydroxybenzotriazole (HOBt) at room temperature for 2 h gave the corresponding amide derivatives. The structures of the target compounds were identified by HRMS, ^1^H-NMR, and ^13^C-NMR spectral analysis.

### 2.2. Biological Results and Discussion

All synthesized compounds were evaluated for their antitumor activity against three cancer cell lines (HeLa, K562 and K562/A02) using the MTT assay. The results are summarized in Table 1.

Some compounds exhibited promising antiproliferative activity against one or more cell lines. Among them, compound **8c** exhibited the most potent antiproliferative activity against HeLa cells (IC_50_ = 1.5 μM), and it showed 14-fold increased activity as compared to the positive control, etoposide (VP-16). Compound **8f** also showed strong potency against HeLa cells with an IC_50_ value of 2.2 μM, which was found to be the second most active compound. Concerning the activity against K562, compounds **8a** and **8b** showed good activities against this cell line with IC_50_ values of 10.1 and 11.0 μM, and they were as effective as VP-16. It is worth noting that seven synthesized compounds (**8a**–**8c**, **8e**, **8f**, **8j** and **8q**) exhibited moderate to good activities against drug-resistant cell line K562/A02, and they were more potent than VP-16. This result indicates that these derivatives possessed a great potential of anti-multidrug resistance.

During the structure-activity relationship studies, we found that compounds containing piperazinyl group (**8a** and **8c**) were more active against the HeLa cell line than those containing benzyl (**8g**–**8j**) and phenethyl (**8l**–**8o**). It was also noted that the substituent on the piperazine ring of the amide derivatives had a remarkable effect on their antiproliferative activity against this cell line. Compound **8c** with the fluorophenyl group on the piperazine ring of the amide derivative showed more potent inhibitory effects than compounds with the methyl, phenyl and nitrophenyl groups on the piperazine ring (**8a**, **8b** and **8d**). Besides the piperazinyl group, other nitrogen-containing heterocyclic amide derivatives and *N*-alkyl amide derivatives were synthesized. The result showed that compounds with a morpholino group (**8e**) and a pyrrolidinocarbonyl group (**8f**) exhibited good to excellent antiproliferative activity against the HeLa cell line, whereas compounds with an alkyl group showed weak or no activity. 

Regarding the activity against the K562 and K562/A02 cell lines, a similar structure-activity relationship was observed. Compound **8a** displayed the best inhibitory activity against K562/A02 cells, indicating that the piperazinyl group of the amide was beneficial to the inhibitory activity. This conclusion was further proved by compounds **8b** and **8c** which also showed good activities. In addition, compound **8e** with the morpholino group and compound **8f** with the pyrrolidinocarbonyl group exhibited an IC_50_ value of around 20 µM against K562 and K562/A02 cells, which suggested that the nitrogen-containing heterocyclic ring of amide derivatives affected the activity. The reason for the above compounds with potential activity might be a new hydrogen bond formation between the amide *N* atom serving as a hydrogen bond receptor and the enzyme serving as hydrogen bond donor. For compounds **8a**–**8c** and **8e**, another possible reason was the introduction of a second heteroatom (*N* or *O*) which might act as another binding site.

## 3. Experimental Section

### 3.1. General Information

NMR spectra were recorded on Bruker AV III 600 NMR spectrometer and Bruker AV 400 instrument (Bruker-Biospin, Karlsruhe, Germany). Solvent signals (CDCl_3_: δ_H_ = 7.26 ppm/δ_C_ = 77.16 ppm; CD_3_COCD_3_: δ_H_ = 2.05 ppm/δ_C_ = 29.84 ppm) were used as reference. High resolution mass spectra (HRMS) were recorded on a Waters SYNAPT G2 HDMS (Waters, Manchester, UK). Reactions were monitored by Thin Layer Chromatography on plates (GF_254_) supplied by Yantai Chemicals (Yantai, China). Neocrotocembraneic acid (resources: extraction from *Croton laevigatus*, purity > 95%, pale yellow solid, mp: 119.6–121.5 °C). Unless otherwise noted, all common reagents and solvents were obtained from commercial suppliers without further purification. ^1^H-NMR and ^13^C-NMR spectra of compounds **8a**–**8r** can be available in Supplementary Materials.

### 3.2. Chemistry

#### General Procedure for the Synthesis of Compounds **8a**–**8r**

To a solution of neocrotocembraneic acid (150 mg, 0.50 mmol) in dry CH_2_Cl_2_ (10 mL) were added HOBt (81 mg, 0.60 mmol) and EDCI (115 mg, 0.60 mmol). The mixture was stirred at room temperature for 2 h, and then the corresponding amines (0.6 mmol 1.2 equiv.) were added. Upon completion, the reaction mixture was washed successively with 1M hydrochloric acid, saturated NaHCO_3_ and brine, then dried over Na_2_SO_4_ and concentrated in vacuo. The residue was purified by column chromatography to give the amide derivatives **8a**–**8r**.

*((1E,5E,9E,11E)-12-Isopropyl-5,9-dimethylcyclotetradeca-1,5,9,11-tetraen-1-yl)(4-methylpiperazin-1-yl)methanone* (**8a**): Yield: 76%; Colorless oil; UV (MeOH) λ_max_ (log ε) 250 (3.9); 1H-NMR (600 MHz, CDCl_3_) δ 6.02 (d, *J* = 11.2 Hz, 1H), 5.95 (d, *J* = 11.2 Hz, 1H), 5.45 (t, *J* = 7.7 Hz, 1H), 5.08 (t, *J* = 6.5 Hz, 1H), 3.64–3.49 (m,4H), 2.45 (t, *J* = 7.7 Hz, 2H), 2.35–2.31 (m, 5H), 2.31–2.24 (m, 7H), 2.22–2.19 (m, 2H), 2.15–2.12 (m, 4H), 1.73 (s, 3H), 1.72 (s, 3H), 1.00 (s, 3H), 0.99 (s, 3H); ^13^C-NMR (150 MHz, CDCl_3_) δ 171.7, 146.6, 135.6, 135.5, 134.7, 133.0, 126.9, 120.2, 118.7, 55.2, 46.2, 38.4, 37.6, 34.2, 28.6, 28.4, 28.3, 25.0, 22.2, 18.2, 18.0; IR ν_max_ (film): 2955, 2925, 2853, 2789, 1623, 1458, 1427, 1291, 1144, 1002 cm^−1^; HRMS *m*/*z* calculated for C_25_H_41_N_2_O [M + H]^+^: 385.3219; found: 385.3219.

*((1E,5E,9E,11E)-12-Isopropyl-5,9-dimethylcyclotetradeca-1,5,9,11-tetraen-1-yl)(4-phenylpiperazin-1-yl)methanone* (**8b**): Yield: 66%; Colorless oil; UV (MeOH) λ_max_ (log ε) 248 (4.3); ^1^H-NMR (600 MHz, CD_3_COCD_3_) δ 7.25–7.22 (m, 2H), 6.99 (dt, *J* = 7.8 Hz, 1.1Hz, 2H), 6.82 (tt, *J* = 7.2 Hz, 1.0Hz, 1H), 6.08 (d, *J* = 11.3 Hz, 1H), 6.06 (d, *J* = 11.3 Hz, 1H), 5.58 (t, *J* = 7.7 Hz, 1H), 5.18–5.15 (m, 1H), 3.68–3.64 (m, 4H), 3.16–3.12 (m, 4H), 2.51 (t, *J* = 7.6 Hz, 2H), 2.37–2.29 (m, 5H), 2.27 (t, *J* = 7.6 Hz, 2H), 2.23–2.19 (m, 4H), 1.79 (s, 3H), 1.74 (s, 3H), 1.01 (s, 3H), 1.00 (s, 3H); ^13^C-NMR (150 MHz, CD_3_COCD_3_) δ 170.6, 151.5, 145.7, 135.4, 134.9, 134.4, 132.9, 128.9, 127.0, 120.2, 119.6, 118.8, 116.3, 49.3, 38.3, 37.0, 33.5, 28.5, 27.7, 27.4, 24.7, 21.5, 17.4, 16.8; IR ν_max_ (film): 3340, 2956, 2921, 2850, 1622, 1599, 1503, 1495, 1435, 1380, 1232, 1155 cm^−1^; HRMS *m*/*z* calculated for C_30_H_43_N_2_O [M + H]^+^: 447.3375; found: 447.3373.

*(4-(4-Fluorophenyl)piperazin-1-yl)((1E,5E,9E,11E)-12-isopropyl-5,9-dimethylcyclotetradeca-1,5,9,11-tetraen-1-yl)methanone* (**8c**): Yield: 71%; Colorless oil; UV (MeOH) λ_max_ (log ε) 242 (4.4); ^1^H-NMR (600 MHz, CD_3_COCD_3_) δ 7.04–6.99 (m, 4H), 6.08 (d, *J* = 11.3 Hz, 1H), 6.05 (d, *J* = 11.3 Hz, 1H), 5.58 (t, *J* = 7.7 Hz, 1H), 5.18–5.14 (m, 1H), 3.68–3.62 (m, 4H), 3.11–3.05 (m, 4H), 2.51 (t, *J* = 7.6 Hz, 2H), 2.37–2.28 (m, 5H), 2.27 (t, *J* = 7.6 Hz, 2H), 2.23–2.18 (m, 4H), 1.78 (s, 3H), 1.73 (s, 3H), 1.01 (s, 3H), 1.00 (s, 3H); ^13^C-NMR (150 MHz, CD_3_COCD_3_) δ 171.5, 157.9 (d, *J* = 235.5 Hz), 149.2, 146.6, 136.3, 135.8, 135.2, 133.8, 127.8, 121.1, 119.6, 119.0 (d, *J* = 7.7 Hz), 116.1 (d, *J* = 22.3 Hz), 51.0, 39.2, 37.9, 34.3, 29.3, 28.5, 28.3, 25.6, 22.4, 18.2, 17.7; IR ν_max_ (film): 2956, 2921, 2850, 1623, 1509, 1433, 1232 cm^−1^; HRMS *m*/*z* calculated for C_30_H_42_FN_2_O [M + H]^+^: 465.3281; found: 465.3283.

*((1E,5E,9E,11E)-12-Isopropyl-5,9-dimethylcyclotetradeca-1,5,9,11-tetraen-1-yl)(4-(4-nitrophenyl)piperazin-1-yl)methanone* (**8d**): Yield: 65%; Colorless oil; UV (MeOH) λ_max_ (log ε) 240 (4.2); ^1^H-NMR (600 MHz, CD_3_COCD_3_) δ 8.12–8.09 (m, 2H), 7.08–7.05 (m, 2H), 6.11–6.06 (m, 2H), 5.63 (t, *J* = 7.7 Hz, 1H), 5.19–5.16 (m, 1H), 3.73–3.69 (m, 4H), 3.55–3.51 (m, 4H), 2.54 (t, *J* = 7.5 Hz, 2H), 2.38–2.30 (m, 5H), 2.29 (t, *J* = 7.6 Hz, 2H), 2.24–2.21 (m, 4H), 1.80 (s, 3H), 1.76 (s, 3H), 1.00 (s, 3H), 0.99 (s, 3H); ^13^C-NMR (150 MHz, CD_3_COCD_3_) δ 170.8, 154.9, 145.8, 138.2, 135.2, 135.1, 134.2, 133.5, 127.1, 125.5, 120.1, 118.7, 112.9, 46.7, 38.3, 36.9, 33.2, 28.6, 27.6, 27.1, 24.8, 21.6, 17.4, 16.7; IR ν_max_ (film): 3435, 2935, 2919, 2847, 1647, 1595, 1496, 1430, 1318, 1237, 1112 cm^−1^; HRMS *m*/*z* calculated for C_30_H_42_N_3_O_3_ [M + H]^+^: 492.3226; found: 492.3233.

*((1E,5E,9E,11E)-12-Isopropyl-5,9-dimethylcyclotetradeca-1,5,9,11-tetraen-1-yl)(morpholino)methanone* (**8e**): Yield: 79%; Colorless oil; UV (MeOH) λ_max_ (log ε) 243 (3.8); ^1^H-NMR (600 MHz, CDCl_3_) δ 6.03 (d, *J* = 11.3 Hz, 1H), 5.97 (d, *J* = 11.3 Hz, 1H), 5.46 (t, *J* = 7.7 Hz, 1H), 5.08–5.04 (m, 1H), 3.64–3.51 (m, 8H), 2.48 (t, *J* = 7.7 Hz, 2H), 2.36–2.28 (m, 3H), 2.28–2.24 (m, 2H), 2.22 (t, *J* = 7.7 Hz, 2H), 2.17–2.11 (m, 4H), 1.73 (s, 3H), 1.72 (s, 3H), 1.01 (s, 3H), 1.00 (s, 3H); ^13^C–NMR (150 MHz, CDCl_3_) δ 171.8, 146.5, 135.7, 135.1, 134.5, 133.7, 127.1, 120.1, 118.7, 67.1, 38.5, 37.5, 33.9, 28.7, 28.1, 28.0, 25.0, 22.3, 18.2, 17.8; IR ν_max_ (film): 3446, 2957, 2922, 2851, 1622, 1457, 1435, 1273, 1115, 1032, 1021 cm^−1^; HRMS *m*/*z* calculated for C_24_H_38_NO_2_ [M + H]^+^: 372.2903; found: 372.2903.

*((1E,5E,9E,11E)-12-Isopropyl-5,9-dimethylcyclotetradeca-1,5,9,11-tetraen-1-yl)(pyrrolidin-1-yl)methanone* (**8f**): Yield: 70%; Colorless oil; UV (MeOH) λ_max_ (log ε) 250 (4.1); ^1^H-NMR (600 MHz, CDCl_3_) δ 6.01 (s, 2H), 5.58 (t, *J* = 7.7 Hz, 1H), 5.09 (td, *J* = 6.6 Hz, 1.3Hz, 1H), 3.46–3.39 (m,4H), 2.51 (t, *J* = 7.7 Hz, 2H), 2.39–2.35 (m, 1H), 2.32–2.29 (m, 2H), 2.28–2.23 (m, 4H), 2.17–2.13 (m, 4H), 1.89–1.81 (m, 4H), 1.74 (s, 3H), 1.72 (s, 3H), 1.00 (s, 3H), 0.99 (s, 3H); ^13^C-NMR (100 MHz, CDCl_3_) δ 171.0, 147.0, 137.4, 135.4, 134.5, 133.5, 127.1, 120.4, 118.3, 38.5, 37.5, 33.6, 28.9, 28.2, 27.4, 25.0, 22.2, 18.2, 17.8; IR ν_max_ (film): 3437, 2957, 2925, 2869, 1648, 1610, 1414, 1189 cm^−1^; HRMS *m*/*z* calculated for C_24_H_38_NO [M + H]^+^: 356.2953; found: 356.2953.

*(1E,5E,9E,11E)-N-Benzyl-12-isopropyl-5,9-dimethylcyclotetradeca-1,5,9,11-tetraenecarboxamide* (**8g**): Yield: 75%; Colorless oil; UV (MeOH) λ_max_ (log ε) 240 (3.6); ^1^H-NMR (600 MHz, CDCl_3_) δ 7.35–7.32 (m, 2H), 7.27–7.25 (m, 3H), 6.01 (d, *J* = 11.3 Hz, 1H), 5.99 (dq, *J* = 11.3 Hz, 1.3 Hz, 1H), 5.88 (t, *J* = 7.5 Hz, 1H), 5.72 (t, *J* = 5.8 Hz, 1H), 5.04 (t, *J* = 6.5 Hz, 1H), 4.38 (d, *J* = 5.7 Hz, 2H), 2.52 (t, *J* = 7.0 Hz, 2H), 2.40–2.35 (m, 1H), 2.34–2.30 (m, 2H), 2.27–2.23 (m, 4H), 2.19–2.17 (m, 2H), 2.10–2.07 (m, 2H), 1.74 (s, 3H), 1.59 (s, 3H), 1.04 (s, 3H), 1.03 (s, 3H); ^13^C-NMR (100 MHz, CDCl_3_) δ 171.5, 146.3, 138.8, 138.8, 136.0, 134.9, 134.0, 128.8, 127.9, 127.6, 127.4, 119.9, 118.9, 43.8, 39.1, 37.1, 33.8, 29.7, 27.6, 26.4, 25.1, 22.3, 18.3, 17.1; IR ν_max_ (film): 3353, 2959, 2925, 2872, 1648, 1525, 1445, 1363, 1272, 1081 cm^−1^; HRMS *m*/*z* calculated for C_27_H_38_NO [M + H]^+^: 392.2953; found: 392.2952.

*(1E,5E,9E,11E)-N-(4-Fluorobenzyl)-12-isopropyl-5,9-dimethylcyclotetradeca-1,5,9,11-tetraenecarboxamide* (**8h**): Yield: 72%; white solid; mp: 103.7–105.6 °C; UV (MeOH) λ_max_ (log ε) 243 (4.2); ^1^H-NMR (600 MHz, CDCl_3_) δ 7.24–7.21 (m, 2H), 7.01 (t, *J* = 8.6 Hz, 2H), 6.01–5.96 (m, 2H), 5.86 (t, *J* = 7.5 Hz, 1H), 5.71 (t, *J* = 5.8 Hz, 1H), 5.04 (t, *J* = 6.7 Hz, 1H), 4.33 (d, *J* = 5.8 Hz, 2H), 2.51 (t, *J* = 7.0 Hz, 2H), 2.39–2.34 (m, 1H), 2.34–2.30 (m, 2H), 2.28–2.23 (m, 4H), 2.19–2.16 (m, 2H), 2.11–2.07 (m, 2H), 1.74 (s, 3H), 1.59 (s, 3H), 1.04 (s, 3H), 1.02 (s, 3H); ^13^C-NMR (100 MHz, CDCl_3_) δ 171.6, 162.2 (d, *J* = 245.4 Hz), 146.3, 138.7, 136.0, 135.1, 134.6 (d, *J* = 3.2 Hz), 133.9, 129.3 (d, *J* = 8.2 Hz), 127.9, 119.9, 118.9, 115.5 (d, *J* = 21.5 Hz), 43.1, 39.1, 37.1, 33.8, 29.7, 27.6, 26.3, 25.1, 22.3, 18.4, 17.1; IR ν_max_ (film): 3370, 2954, 2927, 2868, 1654, 1621, 1510, 1448, 1379, 1222, 1156, 1026 cm^−1^; HRMS *m*/*z* calculated for C_27_H_37_FNO [M + H]^+^: 410.2859; found: 410.2859.

*(1E,5E,9E,11E)-N-(4-Chlorobenzyl)-12-isopropyl-5,9-dimethylcyclotetradeca-1,5,9,11-tetraenecarboxamide* (**8i**): Yield: 69%; white solid; mp: 107.3–109.3 °C; UV (MeOH) λ_max_ (log ε) 242 (4.1); ^1^H-NMR (600 MHz, CDCl_3_) δ 7.30 (d, *J* = 8.4 Hz, 2H), 7.19 (d, *J* = 8.4 Hz, 2H), 6.01–5.97 (m, 2H), 5.85 (t, *J* = 7.5 Hz, 1H), 5.71 (t, *J* = 5.9 Hz, 1H), 5.04 (t, *J* = 6.6 Hz, 1H), 4.33 (d, *J* = 5.9 Hz, 2H), 2.51 (t, *J* = 7.0 Hz, 2H), 2.39–2.35 (m, 1H), 2.34–2.30 (m, 2H), 2.28–2.23 (m, 4H), 2.20–2.17 (m, 2H), 2.12–2.08 (m, 2H), 1.74 (s, 3H), 1.60 (s, 3H), 1.04 (s, 3H), 1.03 (s, 3H); ^13^C-NMR (100 MHz, CDCl_3_) δ 171.6, 146.4, 138.7, 137.4, 136.0, 135.1, 133.9, 133.2, 129.0, 128.9, 128.0, 120.0, 118.9, 43.1, 39.1, 37.2, 33.8, 29.7, 27.6, 26.4, 25.1, 22.3, 18.4, 17.1; IR ν_max_ (film): 3355, 2955, 2922, 2852, 1653, 1614, 1525, 1490, 1091 cm^−1^; HRMS *m*/*z* calculated for C_27_H_37_ClNO [M + H]^+^: 426.2564; found: 426.2565.

*(1E,5E,9E,11E)-N-(4-Bromobenzyl)-12-isopropyl-5,9-dimethylcyclotetradeca-1,5,9,11-tetraenecarboxamide* (**8j**): Yield: 68%; Colorless oil; UV (MeOH) λ_max_ (log ε) 244 (3.9); ^1^H-NMR (600 MHz, CDCl_3_) δ 7.44 (d, *J* = 8.4 Hz, 2H), 7.13 (d, *J* = 8.4 Hz, 2H), 6.01–5.96 (m, 2H), 5.85 (t, *J* = 7.5 Hz, 1H), 5.72 (t, *J* = 6.1 Hz, 1H), 5.04 (t, *J* = 6.8 Hz, 1H), 4.31 (d, *J* = 5.9 Hz, 2H), 2.51 (t, *J* = 7.0 Hz, 2H), 2.39–2.34 (m, 1H), 2.34–2.30 (m, 2H), 2.28–2.23 (m, 4H), 2.19–2.16 (m, 2H), 2.12–2.08 (m, 2H), 1.74 (s, 3H), 1.60 (s, 3H), 1.04 (s, 3H), 1.03 (s, 3H); ^13^C-NMR (150 MHz, CDCl_3_) δ 171.6, 146.3, 138.7, 138.0, 136.0, 135.1, 133.9, 131.8, 129.4, 128.0, 121.2, 120.0, 118.9, 43.2, 39.1, 37.2, 33.8, 29.7, 27.6, 26.4, 25.1, 22.3, 18.4, 17.1; IR ν_max_ (film): 3353, 2958, 2926, 2869, 1732, 1658, 1622, 1515, 1487, 1456, 1265, 1071, 1011 cm^−1^; HRMS *m*/*z* calculated for C_27_H_37_BrNO [M + H]^+^: 470.2059; found: 470.2062.

*(1E,5E,9E,11E)-N-(Furan-2-ylmethyl)-12-isopropyl-5,9-dimethylcyclotetradeca-1,5,9,11-tetraenecarboxamide* (**8k**): Yield: 73%; Colorless oil; UV (MeOH) λ_max_ (log ε) 241 (3.9); ^1^H-NMR (600 MHz, CD_3_COCD_3_) δ 7.44 (dd, *J* = 1.9 Hz, 0.9 Hz, 1H), 6.87 (s, 1H), 6.34 (dd, *J* = 3.2 Hz, 1.9 Hz, 1H), 6.20 (dd, *J* = 3.2 Hz, 0.9 Hz, 1H), 6.11 (t, *J* = 7.7 Hz, 1H), 6.02 (d, *J* = 11.1 Hz, 1H), 5.98 (dq, *J* = 11.1 Hz, 1.5 Hz, 1H), 5.14 (t, *J* = 6.8 Hz, 1H), 4.35 (d, *J* = 5.8 Hz, 2H), 2.46 (t, *J* = 7.6 Hz, 2H), 2.39–2.31 (m, 3H), 2.26–2.21 (m, 4H), 2.18–2.15 (m, 4H), 1.73 (s, 3H), 1.68 (s, 3H), 1.04 (s, 3H), 1.03 (s, 3H); ^13^C-NMR (100 MHz, CDCl_3_) δ 171.4, 151.8, 146.2, 142.1, 138.5, 136.2, 135.2, 133.9, 128.0, 119.8, 118.9, 110.6, 107.2, 39.1, 37.2, 37.0, 33.7, 29.7, 27.5, 26.2, 25.1, 22.3, 18.3, 17.0; IR ν_max_ (film): 3376, 2958, 2926, 2870, 1726, 1661, 1628, 1520, 1507, 1448, 1379, 1190, 1148, 1012 cm^−1^; HRMS *m*/*z* calculated for C_25_H_36_NO_2_ [M + H]^+^: 382.2746; found: 382.2751.

*(1E,5E,9E,11E)-12-Isopropyl-5,9-dimethyl-N-phenethylcyclotetradeca-1,5,9,11-tetraenecarboxamide* (**8l**): Yield: 72%; white solid; mp: 109.5–111.6 °C; UV (MeOH) λ_max_ (log ε) 249 (4.0); ^1^H-NMR (600 MHz, CDCl_3_) δ 7.33 (dd, *J* = 7.6 Hz, 7.4 Hz, 2H), 7.25 (t, *J* = 7.4 Hz, 1H), 7.19 (d, *J* = 7.6 Hz, 2H), 5.98 (d, *J* = 11.3 Hz, 1H), 5.91 (dq, *J* = 11.3 Hz, 1.6 Hz, 1H), 5.67 (t, *J* = 7.5 Hz, 1H), 5.38 (t, *J* = 6.1 Hz, 1H), 4.92 (t, *J* = 6.6 Hz, 1H), 3.44 (q, *J* = 6.6 Hz, 2H), 2.78 (t, *J* = 6.9 Hz, 2H), 2.45 (t, *J* = 7.0 Hz, 2H), 2.36–2.31 (m, 1H), 2.29–2.25 (m, 2H), 2.25–2.21 (m, 2H), 2.19 (t, *J* = 7.0 Hz, 2H), 2.15–2.12 (m, 2H), 2.07–2.03 (m, 2H), 1.70 (s, 3H), 1.65 (s, 3H), 1.02 (s, 3H), 1.01 (s, 3H); ^13^C-NMR (150 MHz, CDCl_3_) δ 171.4, 146.4, 139.4, 139.1, 135.7, 134.4, 133.9, 129.0, 128.8, 127.9, 126.6, 119.9, 118.8, 40.6, 39.2, 37.1, 36.1, 33.6, 29.8, 27.5, 26.2, 25.0, 22.4, 18.5, 17.1; IR ν_max_ (film): 3356, 2955, 2921, 2851, 1658, 1630, 1521, 1454 cm^−1^; HRMS *m*/*z* calculated for C_28_H_40_NO [M + H]^+^: 406.3110; found: 406.3109.

*(1E,5E,9E,11E)-N-(4-Fluorophenethyl)-12-isopropyl-5,9-dimethylcyclotetradeca-1,5,9,11-tetraenecarboxamide* (**8m**): Yield: 58%; Colorless oil; UV (MeOH) λ_max_ (log ε) 243 (4.1); ^1^H-NMR (600 MHz, CDCl_3_) δ 7.15 (dd, *J* = 8.4 Hz, 5.6 Hz, 2H), 7.01 (d, *J* = 8.4 Hz, 2H), 5.98 (d, *J* = 11.3 Hz, 1H), 5.92 (dq, *J* = 11.3 Hz, 1.6 Hz, 1H), 5.68 (t, *J* = 7.4 Hz, 1H), 5.39 (t, *J* = 6.1 Hz, 1H), 4.95 (td, *J* = 6.7 Hz, 1.6 Hz, 1H), 3.40 (q, *J* = 6.7 Hz, 2H), 2.75 (t, *J* = 7.0 Hz, 2H), 2.45 (t, *J* = 7.0 Hz, 2H), 2.36–2.31 (m, 1H), 2.30–2.22 (m, 4H), 2.20 (t, *J* = 7.0 Hz, 2H), 2.16–2.13 (m, 2H), 2.09–2.06 (m, 2H), 1.71 (s, 3H), 1.66 (s, 3H), 1.02 (s, 3H), 1.01 (s, 3H); ^13^C-NMR (150 MHz, CDCl_3_) δ 171.4, 161.7 (d, *J* = 244.4 Hz), 146.4, 139.0, 135.6, 135.0 (d, *J* = 3.3 Hz), 134.5, 133.9, 130.3 (d, *J* = 7.7 Hz), 127.8, 120.0, 118.8, 115.5 (d, *J* = 21.2 Hz), 40.7, 39.2, 37.1, 35.3, 33.6, 29.7, 27.5, 26.2, 25.0, 22.3, 18.4, 17.0; IR ν_max_ (film): 3418, 3366, 2957, 2928, 2870, 1658, 1622, 1510, 1450, 1221 cm^−1^; HRMS *m*/*z* calculated for C_28_H_39_FNO [M + H]^+^: 424.3016; found: 424.3019.

*(1E,5E,9E,11E)-N-(4-Chlorophenethyl)-12-isopropyl-5,9-dimethylcyclotetradeca-1,5,9,11-tetraenecarboxamide* (**8n**): Yield: 55%; Colorless oil; UV (MeOH) λ_max_ (log ε) 221 (4.0); ^1^H-NMR (600 MHz, CDCl_3_) δ 7.29 (d, *J* = 8.4 Hz, 2H), 7.12 (d, *J* = 8.4 Hz, 2H), 5.97 (d, *J* = 11.2 Hz, 1H), 5.92 (dq, *J* = 11.2 Hz, 1.6 Hz, 1H), 5.68 (t, *J* = 7.4 Hz, 1H), 5.37 (t, *J* = 6.1 Hz, 1H), 4.95 (t, *J* = 6.6 Hz, 1H), 3.40 (q, *J* = 6.7 Hz, 2H), 2.75 (t, *J* = 7.0 Hz, 2H), 2.45 (t, *J* = 7.0 Hz, 2H), 2.36–2.31 (m, 1H), 2.30–2.22 (m, 4H), 2.19 (t, *J* = 7.0 Hz, 2H), 2.16–2.13 (m, 2H), 2.09–2.06 (m, 2H), 1.71 (s, 3H), 1.66 (s, 3H), 1.02 (s, 3H), 1.00 (s, 3H); ^13^C-NMR (150 MHz, CDCl_3_) δ 171.4, 146.4, 139.0, 137.9, 135.6, 134.6, 133.9, 132.4, 130.3, 128.8, 127.8, 120.0, 118.8, 40.6, 39.2, 37.1, 35.5, 33.6, 29.7, 27.5, 26.2, 25.0, 22.3, 18.5, 17.0; IR ν_max_ (film): 3367, 2958, 2925, 2853, 1714, 1649, 1517, 1492, 1457, 1368, 1090, 1015 cm^−1^; HRMS *m*/*z* calculated for C_28_H_39_ClNO [M + H]^+^: 440.2720; found: 440.2727.

*(1E,5E,9E,11E)-N-(4-Bromophenethyl)-12-isopropyl-5,9-dimethylcyclotetradeca-1,5,9,11-tetraenecarboxamide* (**8o**): Colorless oil; UV (MeOH) λ_max_ (log ε) 242 (3.9); Yield: 62%; ^1^H-NMR (600 MHz, CDCl_3_) δ 7.44 (d, *J* = 8.4 Hz, 2H), 7.07 (d, *J* = 8.4 Hz, 2H), 5.97 (d, *J* = 11.3 Hz, 1H), 5.92 (dq, *J* = 11.3 Hz, 1.6 Hz, 1H), 5.68 (t, *J* = 7.4 Hz, 1H), 5.37 (t, *J* = 6.1 Hz, 1H), 4.95 (t, *J* = 6.6 Hz, 1H), 3.40 (q, *J* = 6.7 Hz, 2H), 2.74 (t, *J* = 7.0 Hz, 2H), 2.45 (t, *J* = 7.0 Hz, 2H), 2.36–2.31 (m, 1H), 2.30–2.23 (m, 4H), 2.20 (t, *J* = 7.0 Hz, 2H), 2.17–2.13 (m, 2H), 2.09–2.06 (m, 2H), 1.71 (s, 3H), 1.66 (s, 3H), 1.02 (s, 3H), 1.01 (s, 3H); ^13^C-NMR (150 MHz, CDCl_3_) δ 171.4, 146.4, 139.0, 138.4, 135.7, 134.6, 133.9, 131.8, 130.7, 127.9, 120.5, 120.0, 118.8, 40.5, 39.2, 37.1, 35.6, 33.6, 29.8, 27.5, 26.2, 25.0, 22.3, 18.5, 17.0; IR ν_max_ (film): 3418, 2948, 2922, 2843, 1648, 1448, 1032, 1017 cm^−1^; HRMS *m*/*z* calculated for C_28_H_39_BrNO [M + H]^+^: 484.2215; found: 484.2208.

*(1E,5E,9E,11E)-N-Cyclopentyl-12-isopropyl-5,9-dimethylcyclotetradeca-1,5,9,11-tetraenecarboxamide* (**8p**): Yield: 80%; white solid; mp: 106.2–107.9 °C; UV (MeOH) λ_max_ (log ε) 249 (4.2); ^1^H-NMR (600 MHz, CD_3_COCD_3_) δ 6.22 (d, *J* = 7.1 Hz, 1H), 6.05–6.01 (m, 3H), 5.16 (t, *J* = 6,8 Hz, 1H), 4.14–4.06 (m, 1H), 2.43 (t, *J* = 7.5 Hz, 2H), 2.39–2.33 (m, 1H), 2.32–2.28 (m, 2H), 2.27–2.24 (m, 2H), 2.22 (t, *J* = 7.6 Hz, 2H), 2.19–2.13 (m, 4H), 1.93–1.85 (m, 2H), 1.73 (s, 3H), 1.72 (s, 3H), 1.68–1.62 (m, 2H), 1.58–1.51 (m, 2H), 1.44–1.37 (m, 2H), 1.05 (s, 3H), 1.03 (s, 3H); ^13^C-NMR (150 MHz, CD_3_COCD_3_) δ 169.9, 146.5, 139.4, 135.7, 135.2, 134.0, 128.3, 121.1, 119.7, 51.8, 39.5, 38.0, 34.9, 33.4, 29.2, 27.3, 25.6, 24.5, 22.4, 18.3, 17.2; IR ν_max_ (film): 3408, 2954, 2928, 2868, 1652, 1617, 1533, 1456, 1018 cm^−1^; HRMS *m*/*z* calculated for C_25_H_40_NO [M + H]^+^: 370.3110; found: 370.3099.

*(1E,5E,9E,11E)-12-Isopropyl-5,9-dimethyl-N-(prop-2-yn-1-yl)cyclotetradeca-1,5,9,11-tetraenecarboxamide* (**8q**): Yield: 48%; Colorless oil; UV (MeOH) λ_max_ (log ε) 249 (3.9); ^1^H-NMR (600 MHz, CDCl_3_) δ 6.03 (d, *J* = 11.3 Hz, 1H), 5.98 (dq, *J* = 11.3 Hz, 1.5 Hz, 1H), 5.81 (t, *J* = 7.4 Hz, 1H), 5.46 (t, *J* = 5.0 Hz, 1H), 5.07 (t, *J* = 6.7 Hz, 1H), 3.93 (d, *J* = 5.2 Hz, 2.6 Hz, 2H), 2.49 (t, *J* = 6.8 Hz, 2H), 2.38–2.34 (m, 1H), 2.33–2.27 (m, 4H), 2.24 (t, *J* = 6.8 Hz, 2H), 2.22 (t, *J* = 2.6 Hz, 1H), 2.21–2.18 (m, 4H), 1.76 (s, 6H), 1.03 (s, 3H), 1.02 (s, 3H); ^13^C-NMR (150 MHz, CDCl_3_) δ 171.4, 146.2, 138.3, 136.2, 135.6, 133.7, 128.1, 119.9, 118.9, 80.1, 71.6, 39.1, 37.2, 33.5, 29.9, 29.7, 27.1, 26.0, 25.1, 22.3, 18.6, 16.9; IR ν_max_ (film): 3357, 3311, 2955, 2920, 2851, 1658, 1631, 1507, 1448 cm^−1^; HRMS *m*/*z* calculated for C_23_H_34_NO [M + H]^+^: 340.2640; found: 340.2634.

*(1E,5E,9E,11E)-N-Isobutyl-12-isopropyl-5,9-dimethylcyclotetradeca-1,5,9,11-tetraenecarboxamide* (**8r**): Yield: 51%; Colorless oil; UV (MeOH) λ_max_ (log ε) 241 (3.6); ^1^H-NMR (600 MHz, CDCl_3_) δ 6.03–5.99 (m, 2H), 5.80 (t, *J* = 7.4 Hz, 1H), 5.47 (t, *J* = 6.2 Hz, 1H), 5.07 (t, *J* = 6.8 Hz, 1H), 3.00 (t, *J* = 6.5 Hz, 2H), 2.47 (t, *J* = 7.1 Hz, 2H), 2.37–2.33 (m, 1H), 2.32–2.26 (m, 4H), 2.23 (t, *J* = 7.1 Hz, 2H), 2.19–2.14 (m, 5H), 1.74 (s, 3H), 1.72 (s, 3H), 1.03 (s, 3H), 1.02 (s, 3H), 0.90 (s, 3H), 0.89 (s, 3H); ^13^C-NMR (100 MHz, CDCl_3_) δ 171.5, 146.5, 139.3, 135.8, 134.2, 134.1, 127.9, 120.1, 118.9, 46.9, 39.2, 37.3, 33.8, 29.7, 28.8, 27.7, 26.4, 25.2, 22.3, 20.3, 18.5, 17.2; IR ν_max_ (film): 3391, 2958, 2930, 2871, 1652, 1539, 1457, 1386, 1022 cm^−1^; HRMS *m*/*z* calculated for C_24_H_40_NO [M + H]^+^: 358.3110; found: 358.3103.

### 3.3. Evaluation of the Biological Activity

The antiproliferative activity of compounds **8a**–**8r** was evaluated with human cervical cancer cell line (HeLa), chronic myeloid leukemia cell line (K562) and leukemia multidrug-resistant cell line (K562/A02) by the MTT method in vitro, with etoposide (VP-16) as positive control. The three tumor cell lines were cultured in RPMI-1640 containing 10% FBS, 2 mmol∙L^−1^ glutamine, 100 U∙mL^−1^ penicillin, and 100 µg∙mL^−1^ streptomycin at 37 °C in a humidified atmosphere with 5% CO_2_. The cells were seeded at a density of 5 × 10^3^ cells/well in 96-well plates and allowed to attach for 24 h. The thiazolyl blue tetrazolium bromide (MTT) assay was performed to quantify cell viability following treatment with the synthetic compounds or reference compound etoposide (VP-16). After 48 h, 20 μL MTT (5 mg∙mL^−1^) solution was added for 4 h at 37 °C. Then, the supernatant was discarded and dimethylsulfoxide (150 μL) was added to dissolve the formazan product. The intensity was measured at a wavelength of 490 nm.

## 4. Conclusions

In summary, a new series of amide derivatives of neocrotocembraneic acid were designed, synthesized and evaluated for their anticancer activity against three human cancer cell lines (HeLa, K562 and K562/A02). Some of the synthesized compounds showed potent antiproliferative activity against one or more cell lines. Particularly, compound **8a** exhibited promising activity against all three cancer cell lines, including drug-resistant cell line K562/A02. Preliminary SAR analysis showed that the piperazinyl group of the amide derivatives had a significant impact on the anticancer activity.

## Figures and Tables

**Table 1:** Cytotoxic activities of the synthesized derivatives against Hela, K562 and K562/A02.

Compound	IC_50_ (μM) ^a,b^			RF
	HeLa	K562	K562/A02	
**8a**	11.0	10.1	10.9	1.08
**8b**	44.7	11.0	19.4	1.76
**8c**	1.5	21.0	27.9	1.33
**8d**	>100	-	-	-
**8e**	15.9	21.1	21.3	1.01
**8f**	2.2	18.3	19.1	1.04
**8g**	>100	-	-	-
**8h**	>100	-	-	-
**8i**	>100	-	-	-
**8j**	62.2	18.0	19.9	1.11
**8k**	>100	-	-	-
**8l**	40.6	>100	>100	-
**8m**	>100	>100	>100	-
**8n**	26.7	65.8	85.1	1.29
**8o**	>100	-	-	-
**8p**	33.7	>100	>100	-
**8q**	65.7	22.4	26.8	1.20
**8r**	>100	-	-	-
neocrotocem-braneic acid	45.4	-	-	-
VP-16	21.1	8.2	35.1	4.3

^a^ The value is the average of three replicates; ^b^ IC_50_: concentration that causes a 50% reduction of cell growth. “-”: not evaluated. RF: resistance factor was calculated from the ratio of the growth inhibition constant (IC_50_) of the resistant cell sub-line to that of the parental cell line. VP-16: etoposide—the clinically used anticancer drugs.

## Supplementary Materials

The following are available online at http://www.mdpi.com/1420-3049/21/11/1581/s1.
