# Supplementary material for: Semisynthetic and SAR Studies of Amide Derivatives of Neocrotocembraneic Acid as Potential Antitumor Agents
Source: Molecules. 2016 Nov 19;21(11):1581. doi: 10.3390/molecules21111581 (PMC6273784; doi:10.3390/molecules21111581)
Supplement: Supplementary file 1 [file molecules-21-01581-s001.pdf]

Supplementary Materials: Semisynthetic and SAR Studies of Amide Derivatives of Neocrotocembraneic Acid as Potential Antitumor Agents

Hai Shang, Ling-Yu Li, Wei-Hua Cheng, Jun Luo, Hong-Wu Zhang and Zhong-Mei Zou

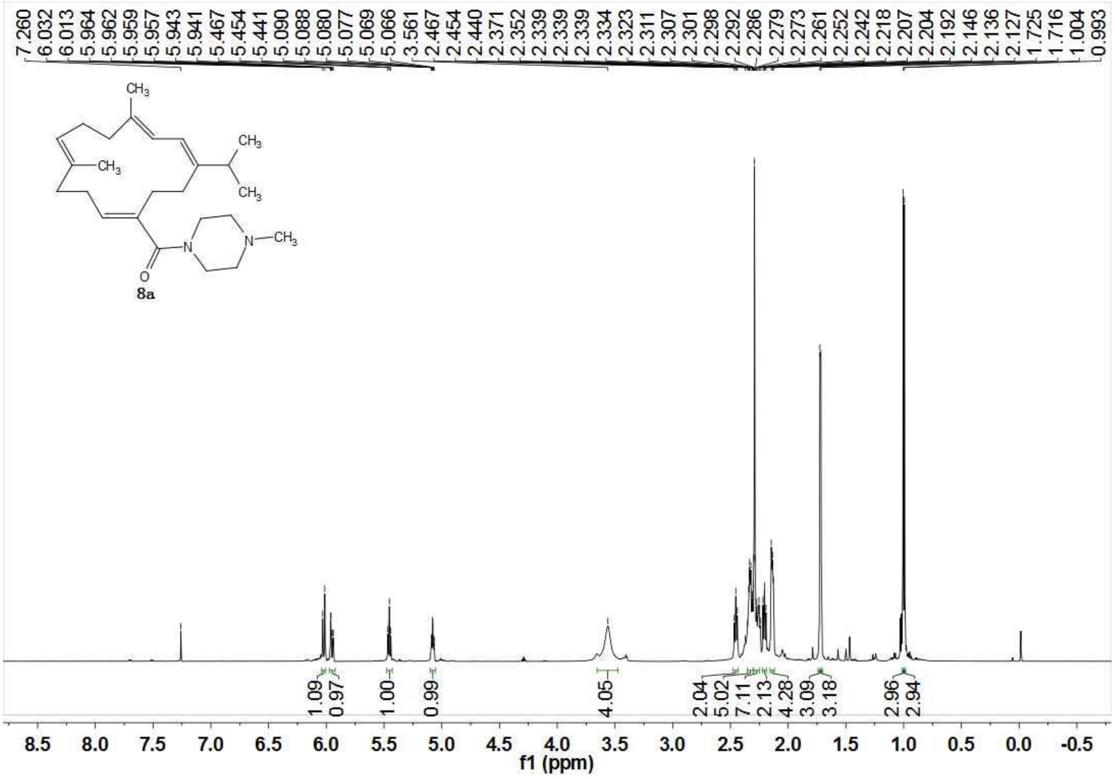

Figure S1. ^{1}H-NMR Spectrum for **8a** ($CDCl_3$, 600 MHz).

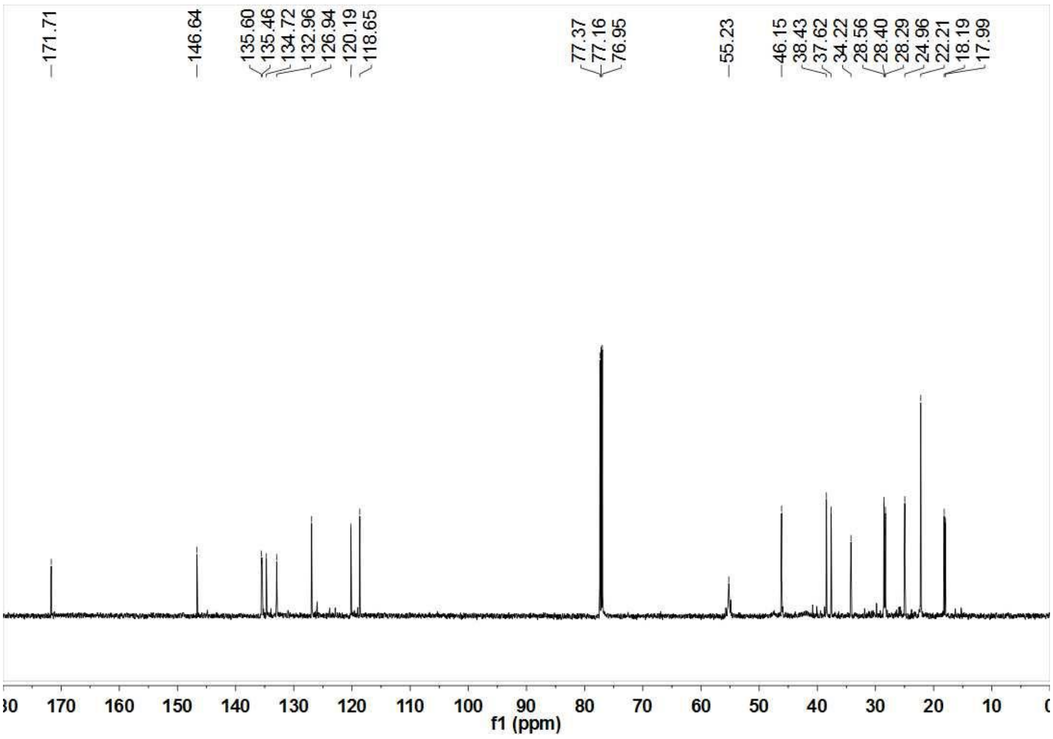

Figure S2. ^{13}C-NMR Spectrum for **8a** ($CDCl_3$, 150 MHz).

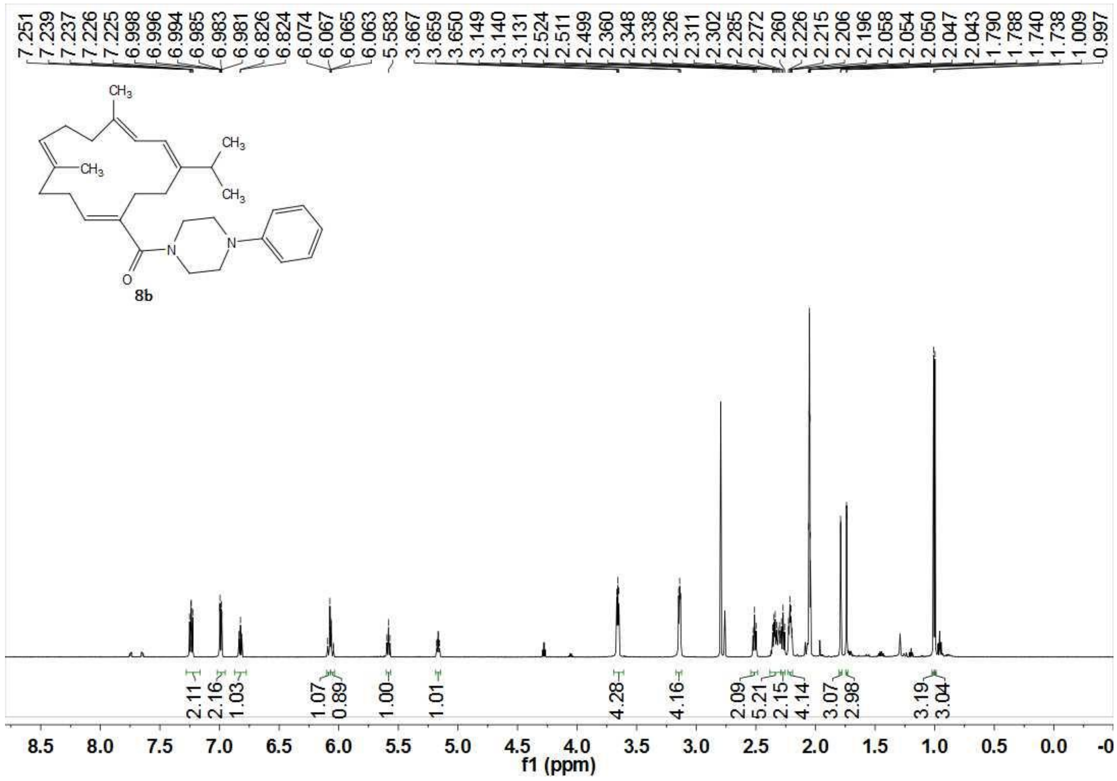

Figure S3. ^{1}H-NMR Spectrum for **8b** (CD_3COCD_3, 600 MHz).

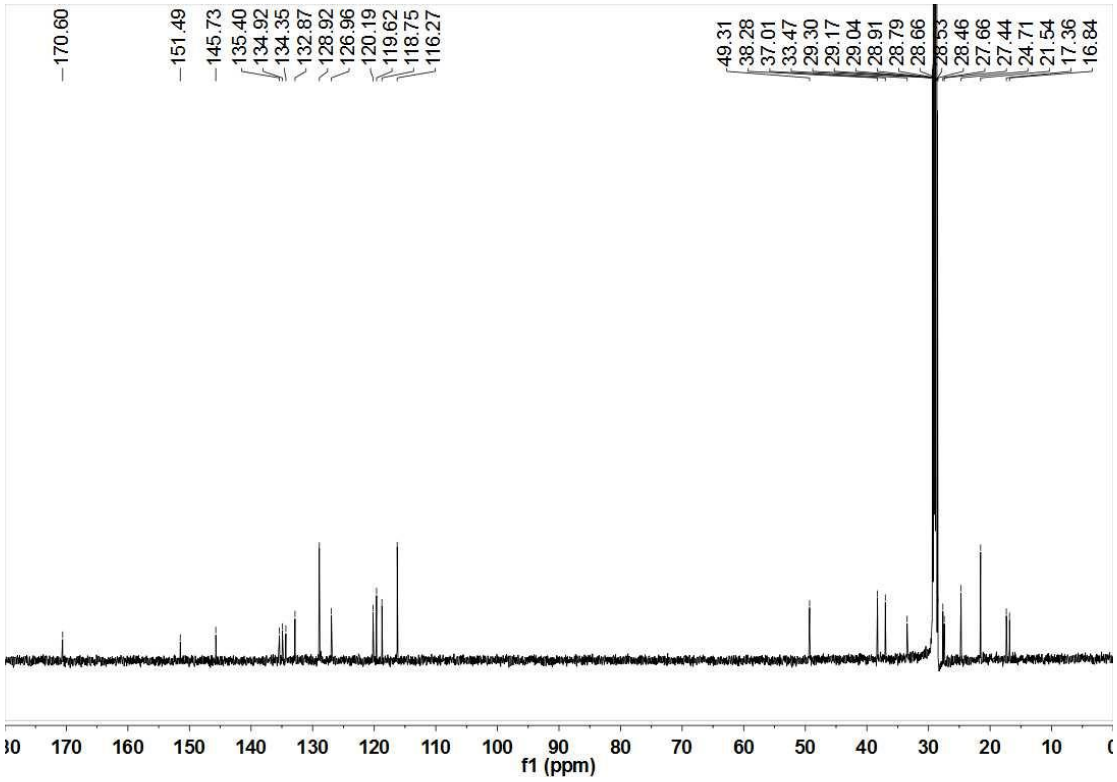

Figure S4. ^{13}C-NMR Spectrum for **8b** (CD_3COCD_3, 150 MHz).

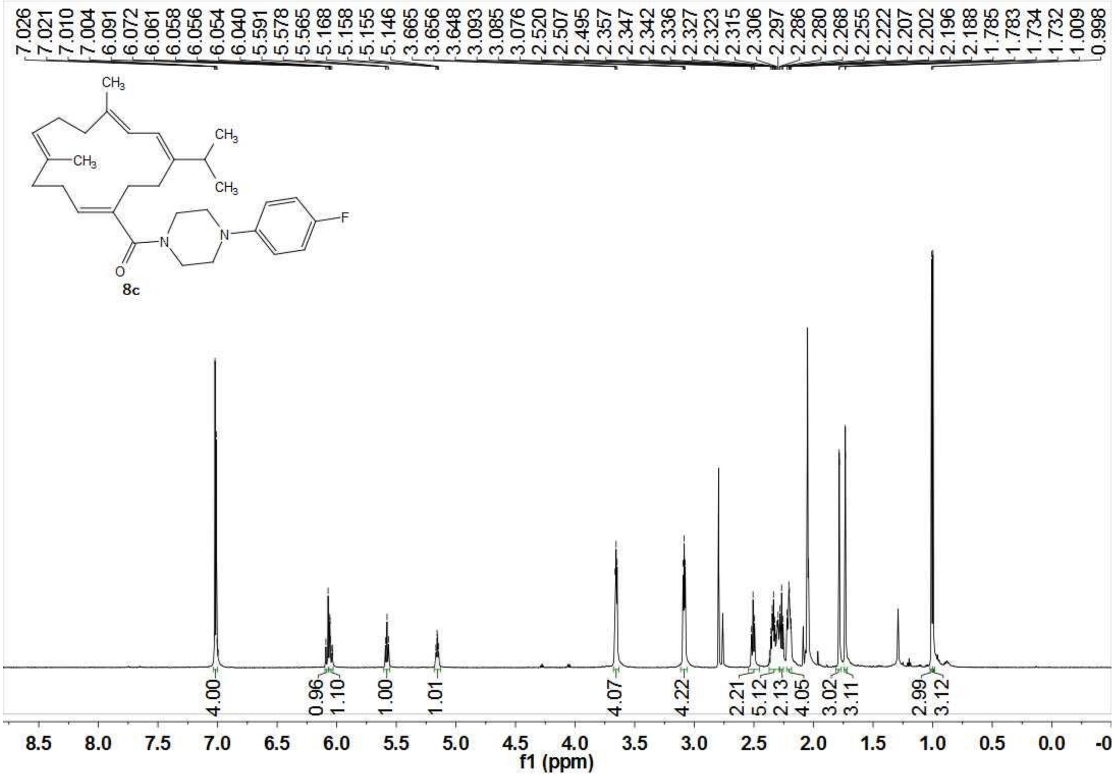

Figure S5. ^{1}H-NMR Spectrum for **8c** (CD_3COCD_3, 600 MHz).

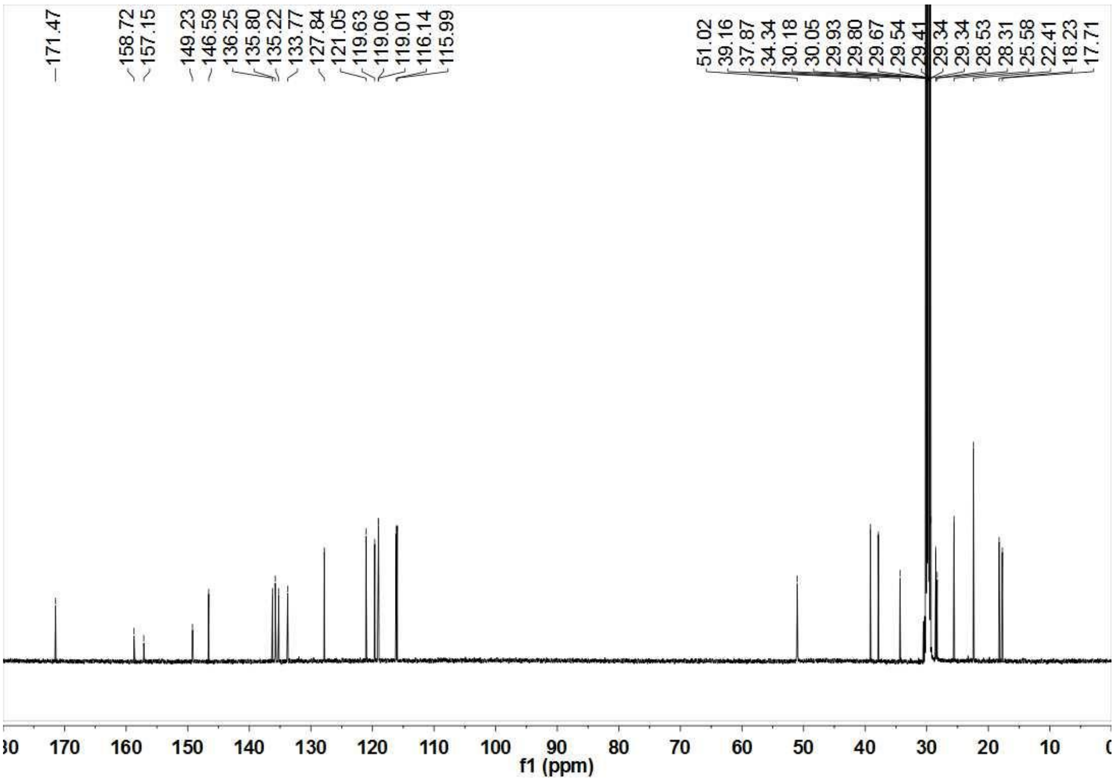

Figure S6. ^{13}C-NMR Spectrum for **8c** (CD_3COCD_3, 150 MHz).

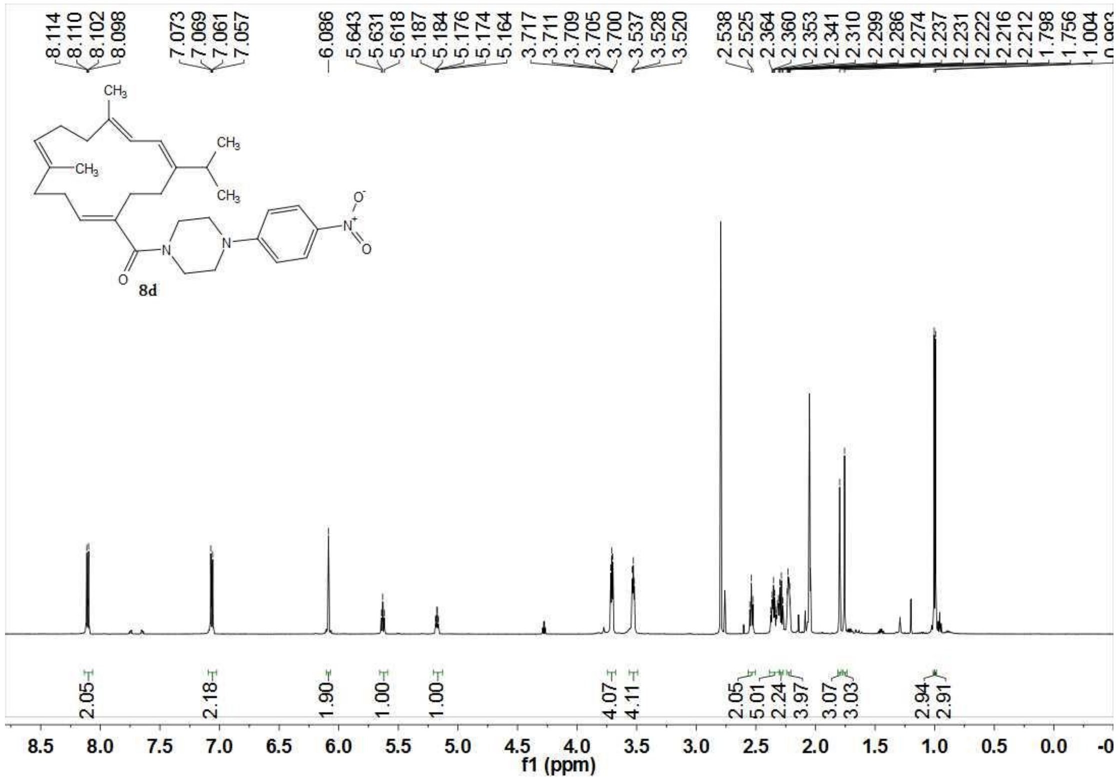

Figure S7. ^{1}H-NMR Spectrum for **8d** (CD_3COCD_3, 600 MHz).

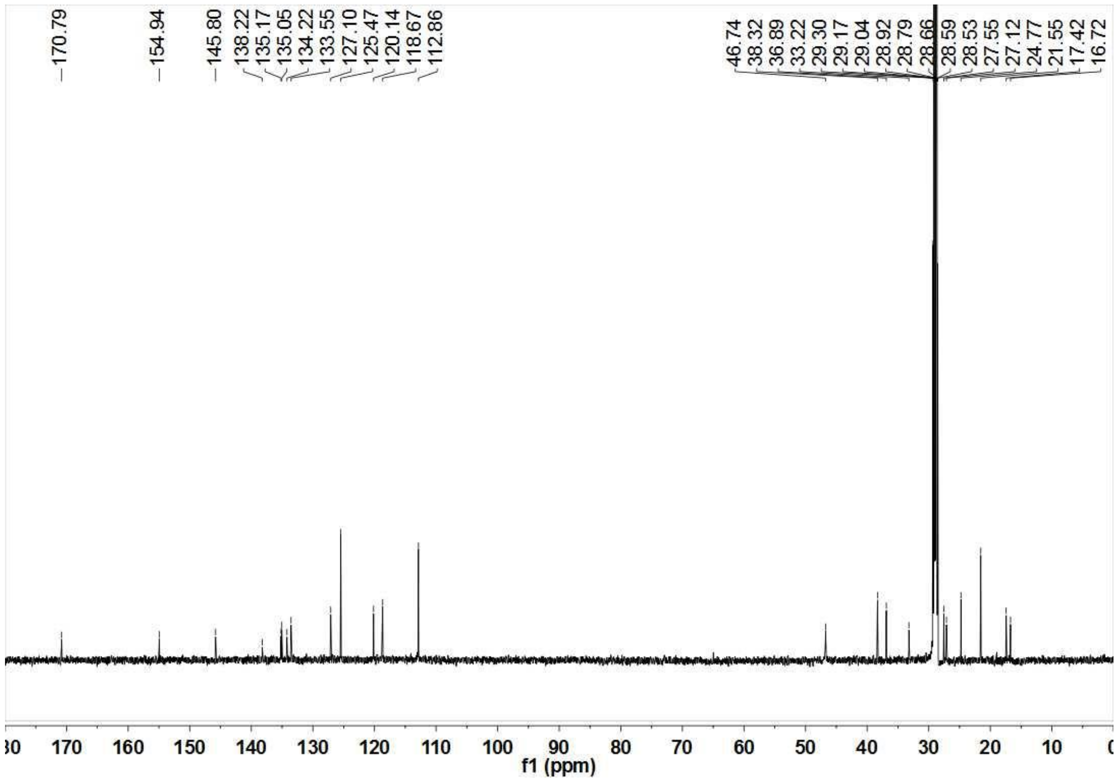

Figure S8. ^{13}C-NMR Spectrum for **8d** (CD_3COCD_3, 150 MHz).

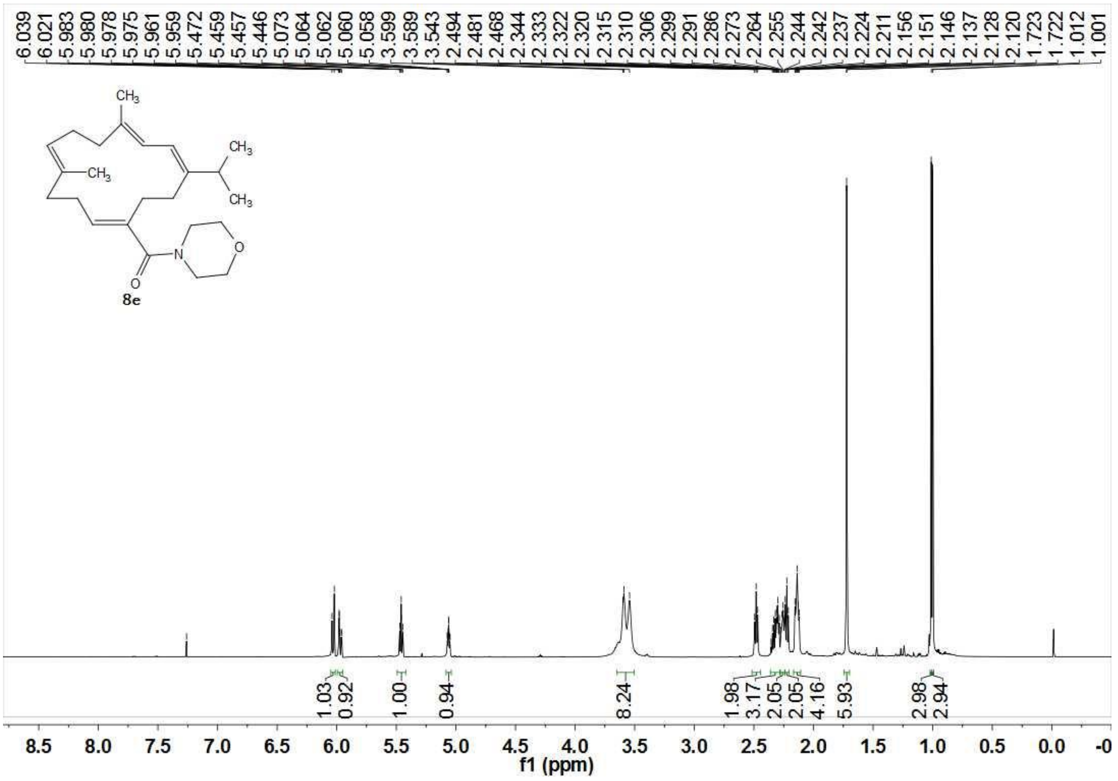

Figure S9. ^{1}H-NMR Spectrum for **8e** ($CDCl_3$, 600 MHz).

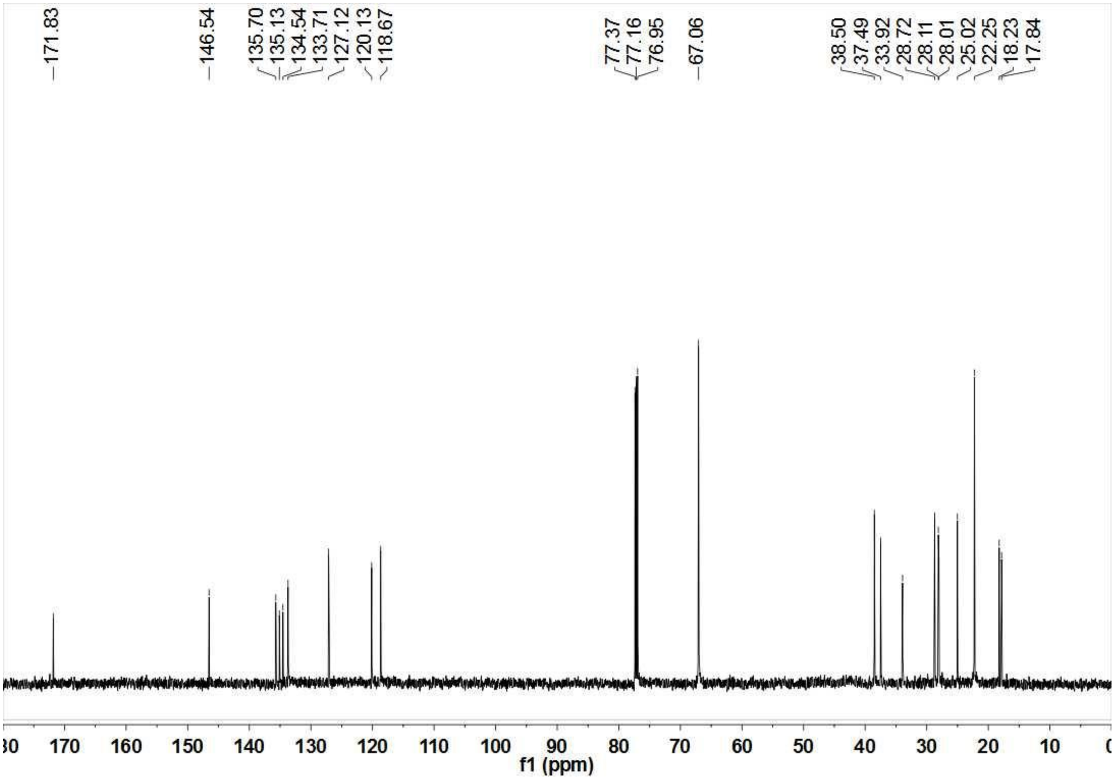

Figure S10. ^{13}C-NMR Spectrum for **8e** ($CDCl_3$, 150 MHz).

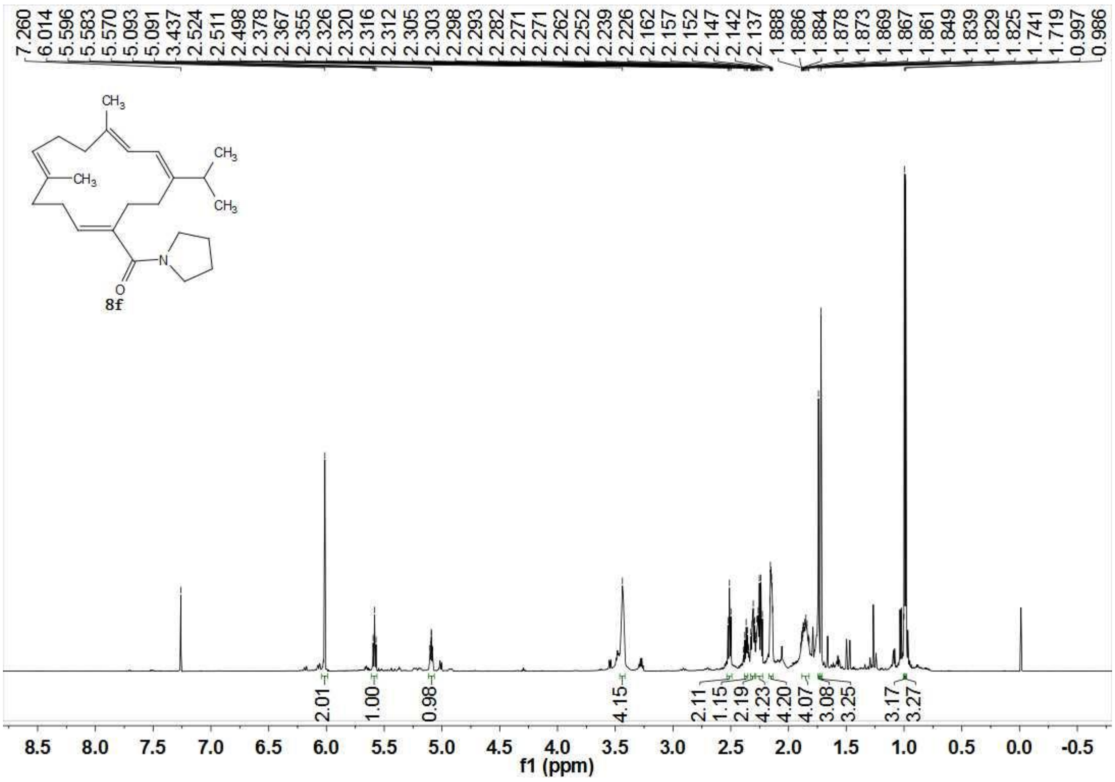

Figure S11. ^{1}H-NMR Spectrum for **8f** ($CDCl_3$, 600 MHz).

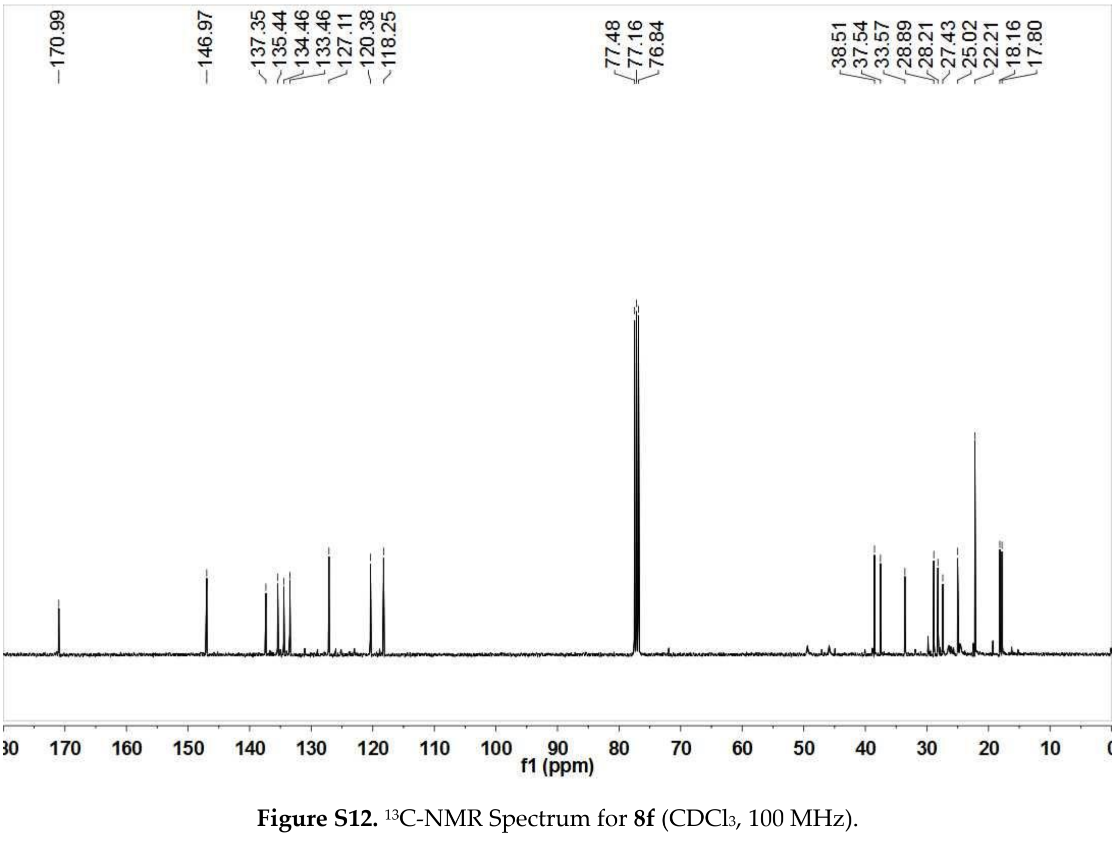

Figure S12. ^{13}C-NMR Spectrum for **8f** ($CDCl_3$, 100 MHz).

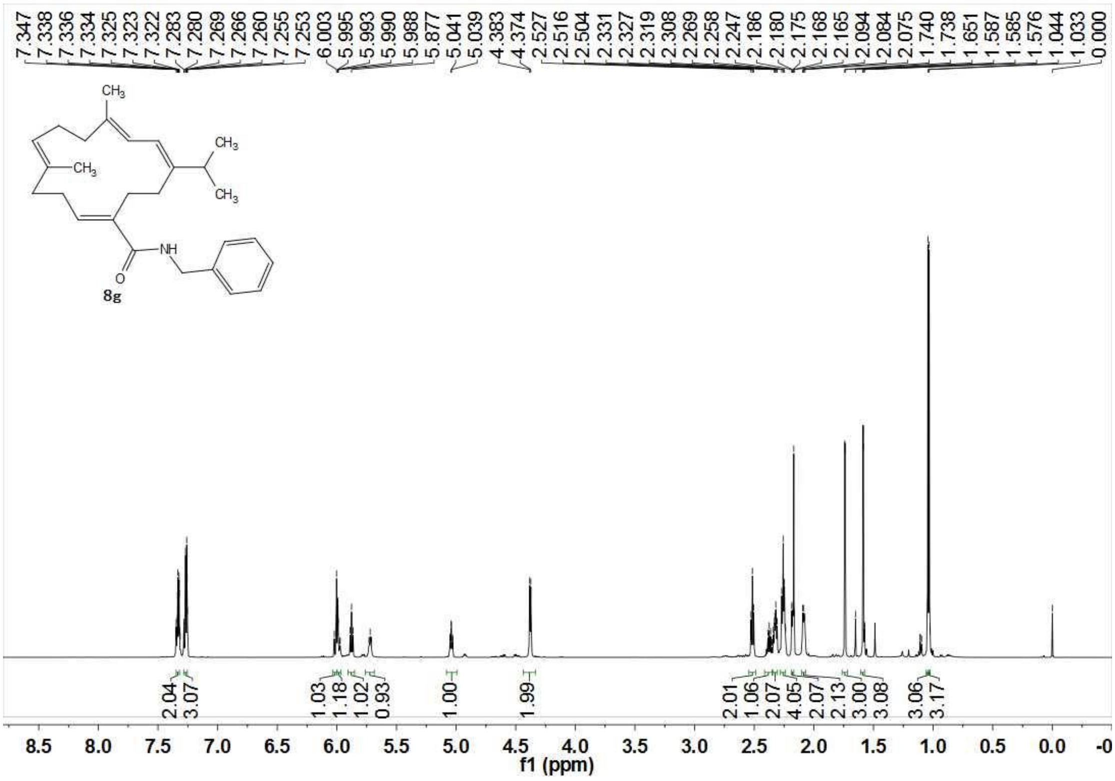

Figure S13. ^{1}H-NMR Spectrum for **8g** ($CDCl_3$, 600 MHz).

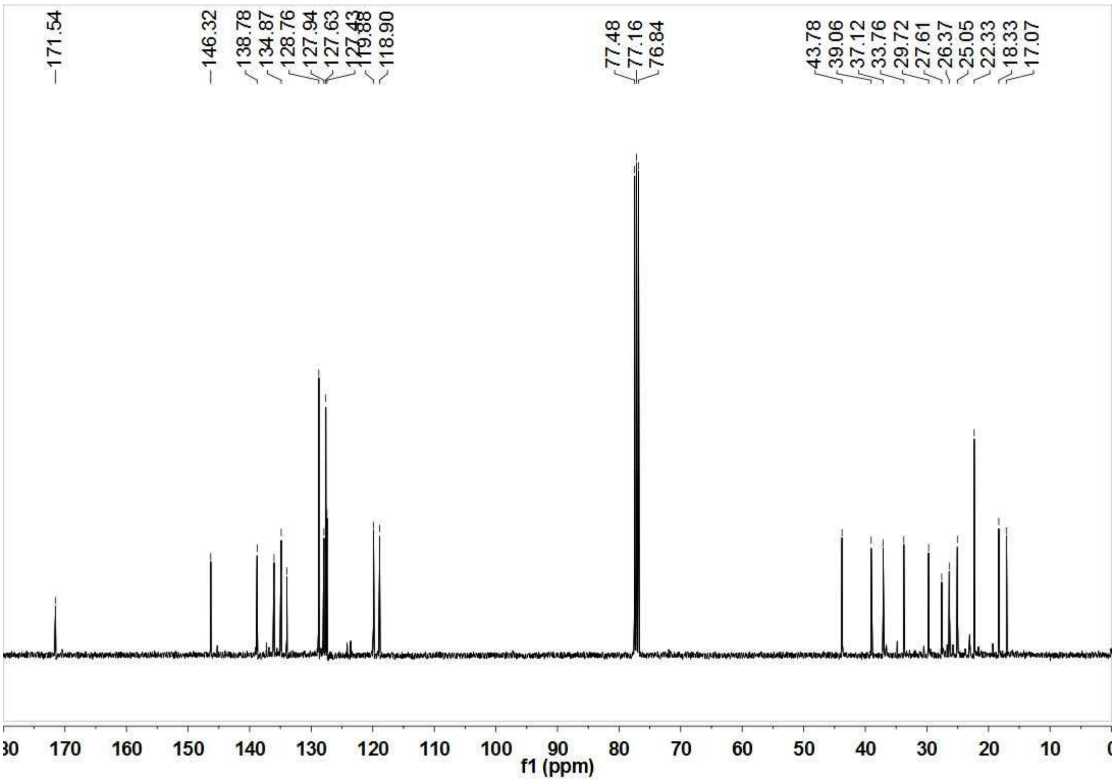

Figure S14. ^{13}C-NMR Spectrum for **8g** ($CDCl_3$, 100 MHz).

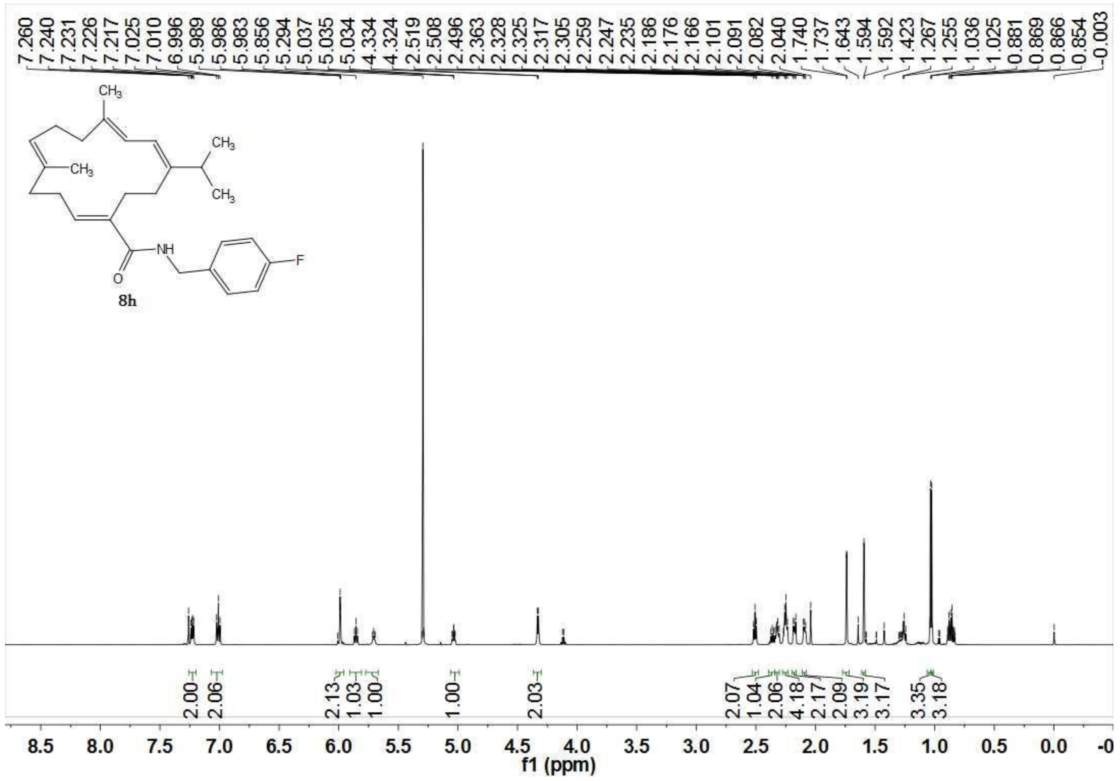

Figure S15. ^{1}H-NMR Spectrum for **8h** ($CDCl_3$, 600 MHz).

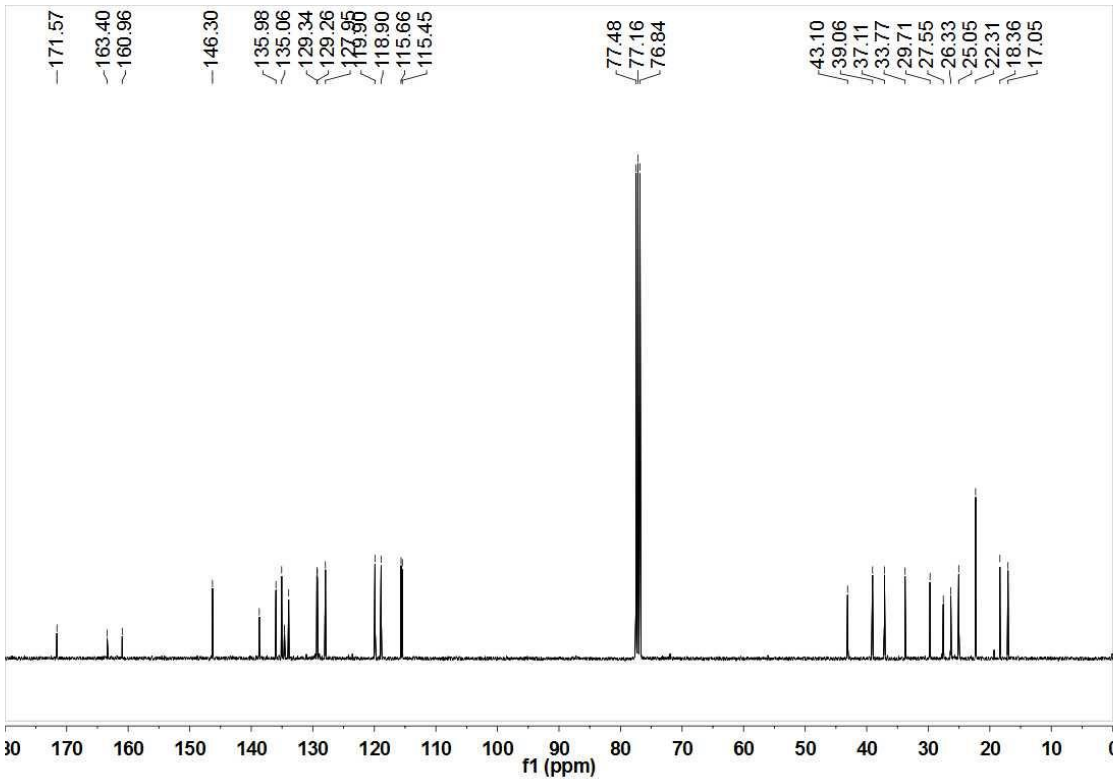

Figure S16. ^{13}C-NMR Spectrum for **8h** ($CDCl_3$, 100 MHz).

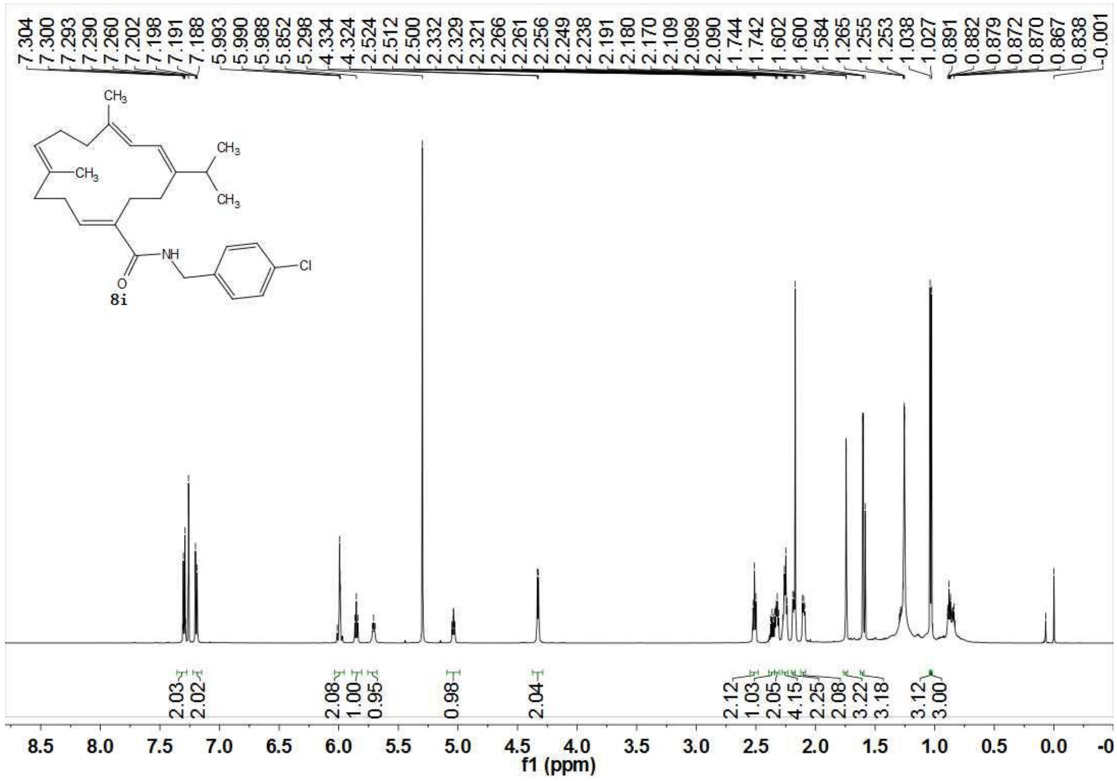

Figure S17. ^{1}H-NMR Spectrum for **8i** ($CDCl_3$, 600 MHz).

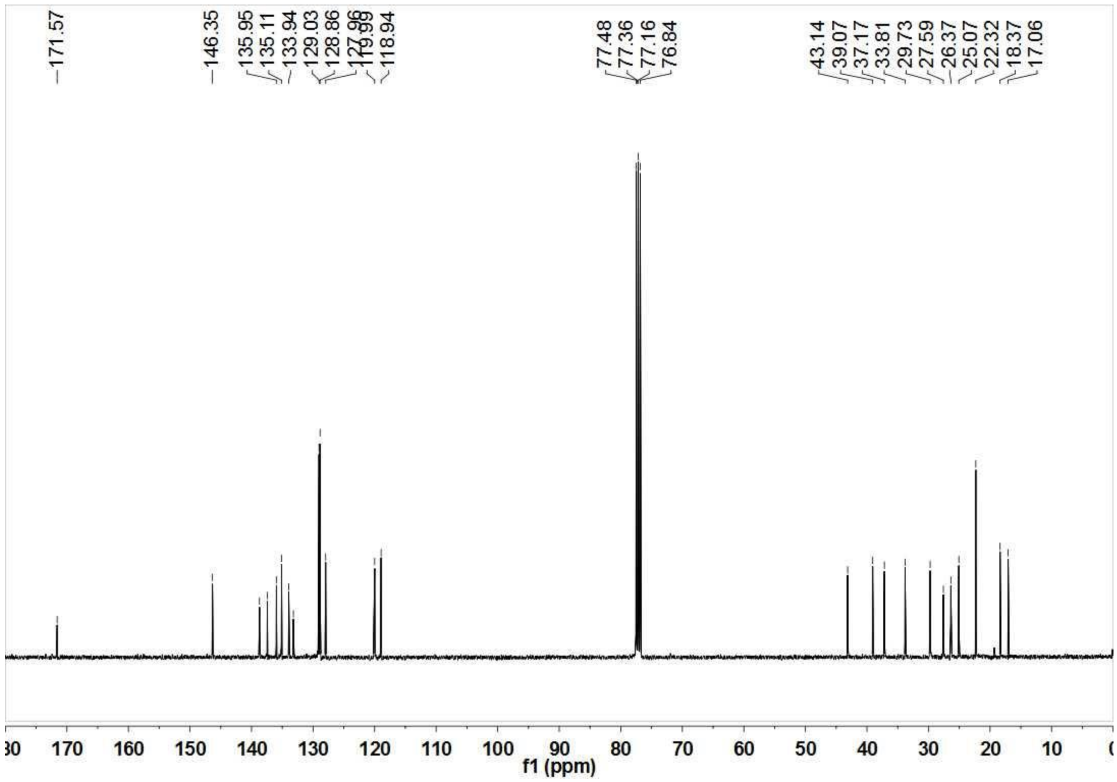

Figure S18. ^{13}C-NMR Spectrum for **8i** ($CDCl_3$, 100 MHz).

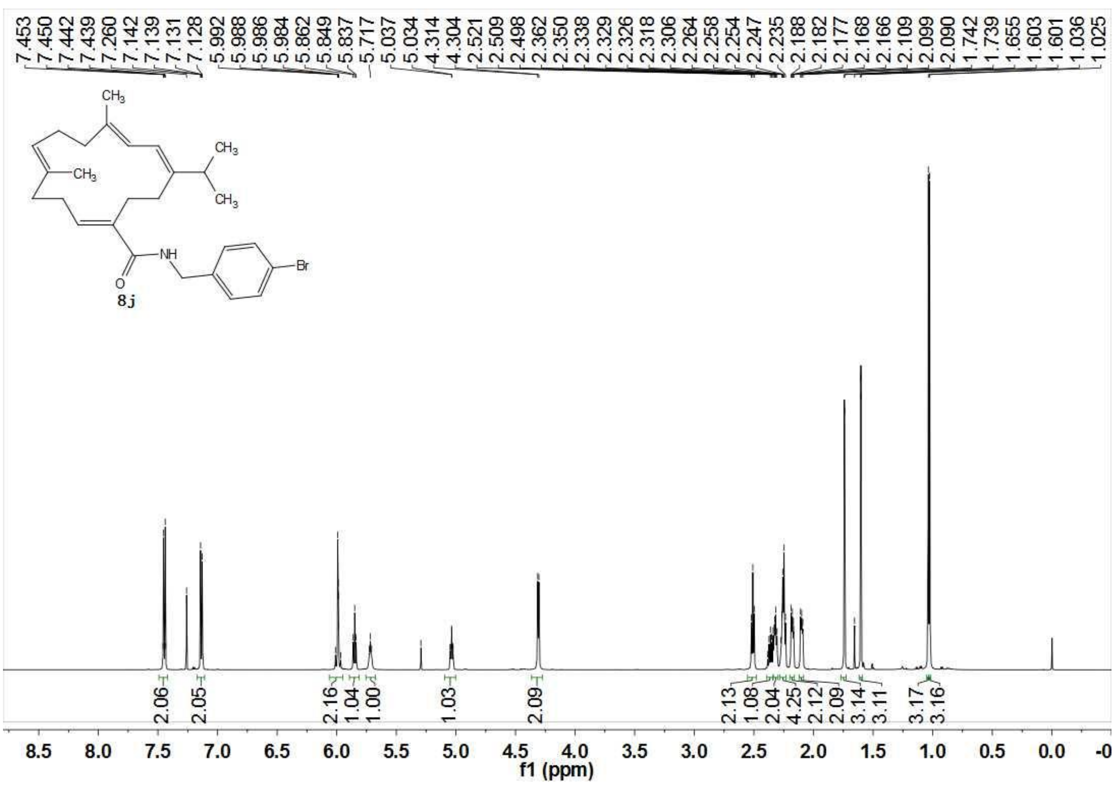

Figure S19. ^{1}H-NMR Spectrum for **8j** ($CDCl_3$, 600 MHz).

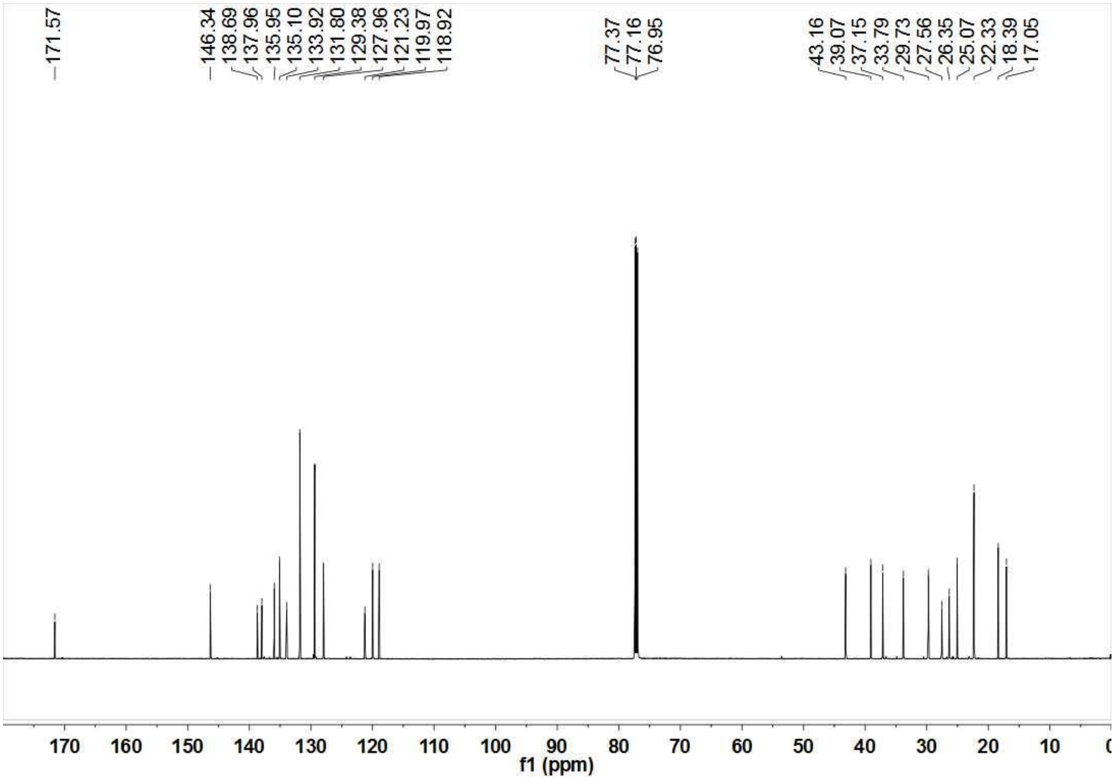

Figure S20. ^{13}C-NMR Spectrum for **8j** ($CDCl_3$, 150 MHz).

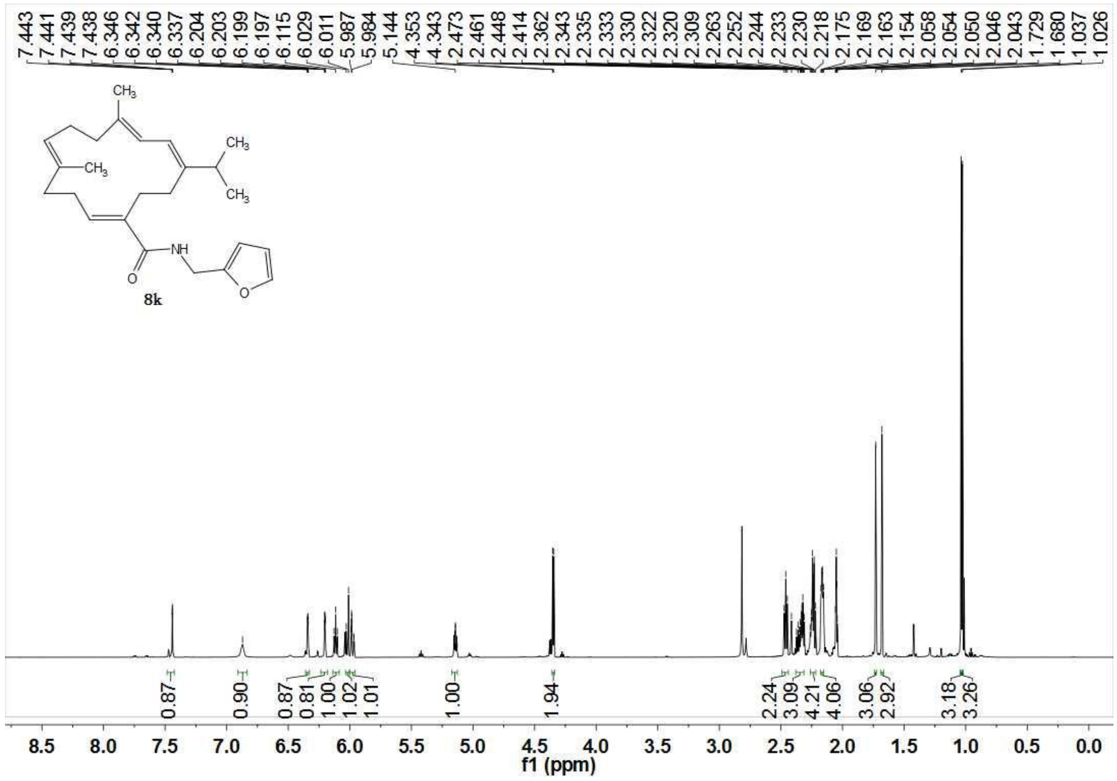

Figure S21. ^{1}H-NMR Spectrum for **8k** (CD_3COCD_3, 600 MHz).

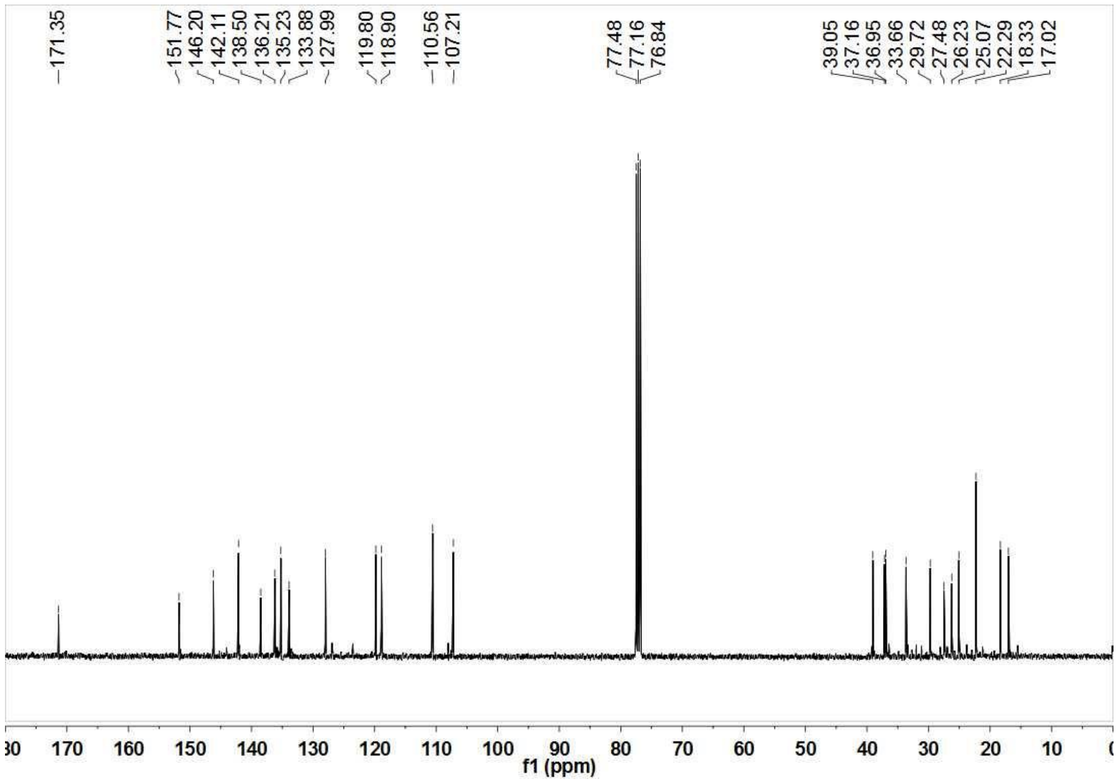

Figure S22. ^{13}C-NMR Spectrum for **8k** ($CDCl_3$, 100 MHz).

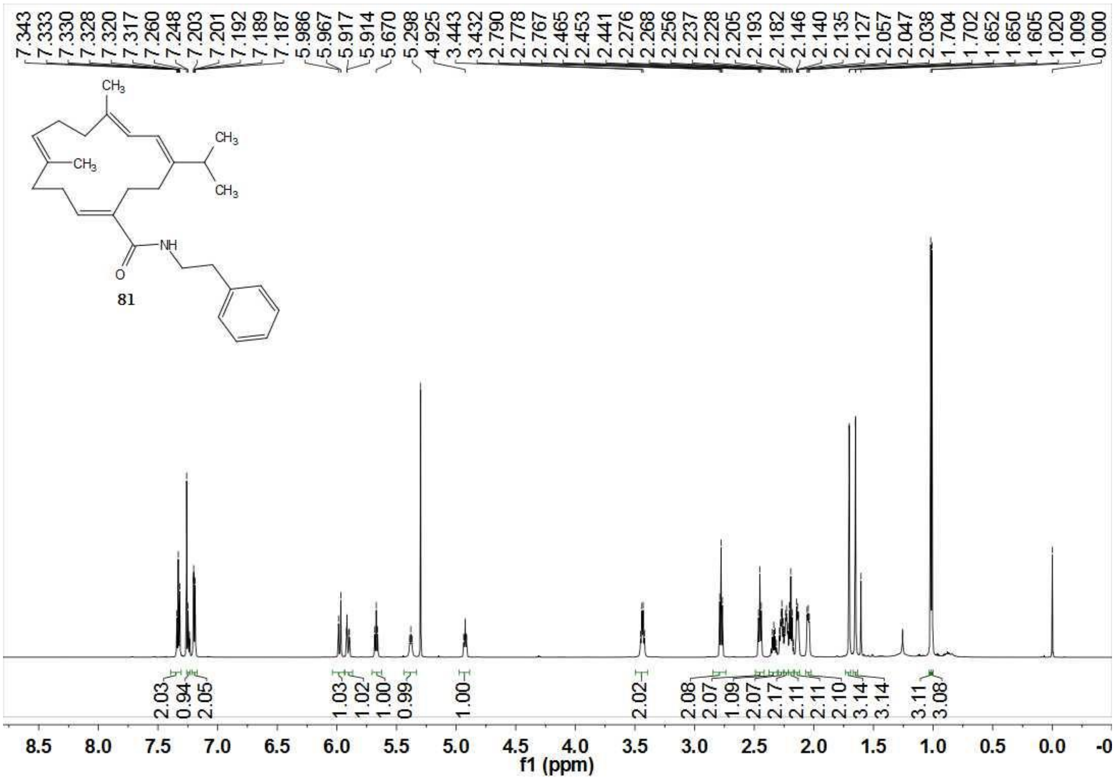

Figure S23. ^{1}H-NMR Spectrum for **8l** ($CDCl_3$, 600 MHz).

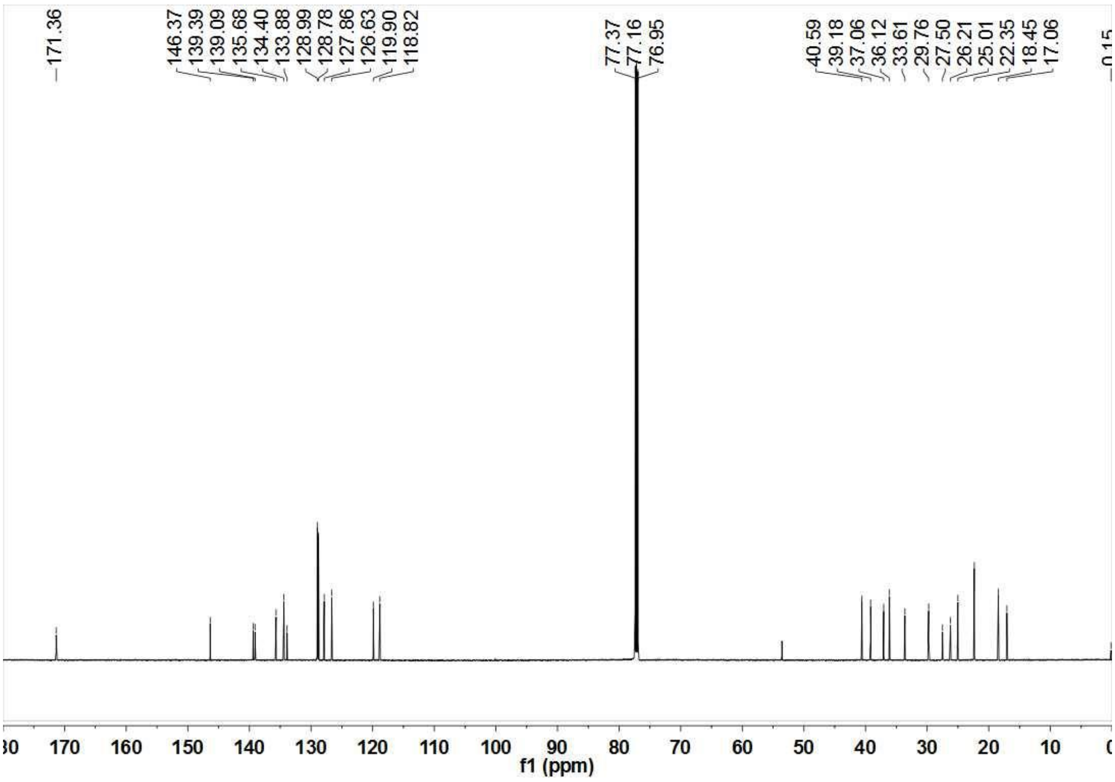

Figure S24. ^{13}C-NMR Spectrum for **8l** ($CDCl_3$, 150 MHz).

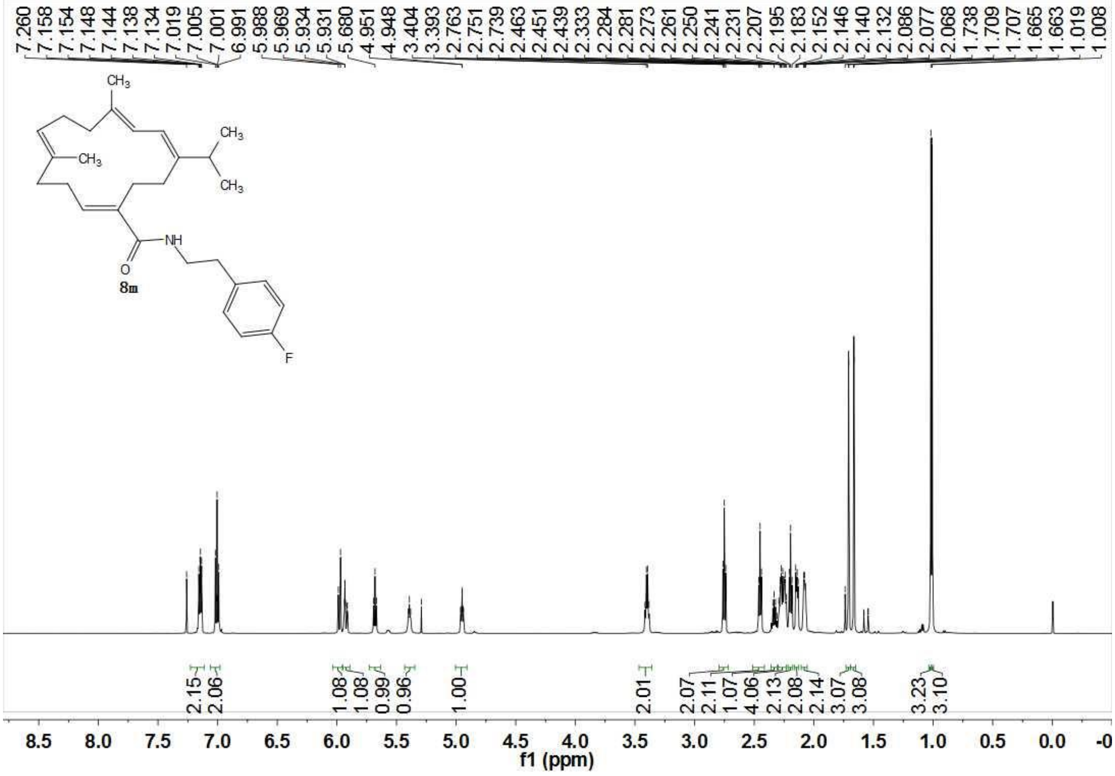

Figure S25. ^{1}H-NMR Spectrum for **8m** ($CDCl_3$, 600 MHz).

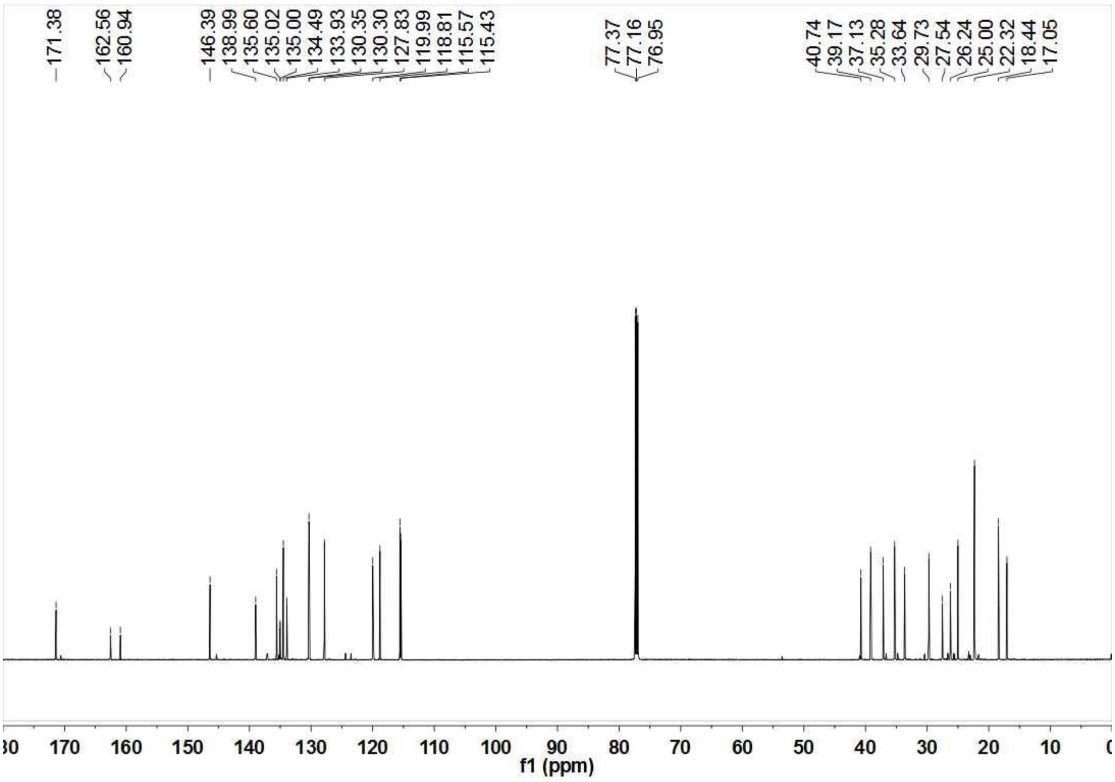

Figure S26. ^{13}C-NMR Spectrum for **8m** ($CDCl_3$, 150 MHz).

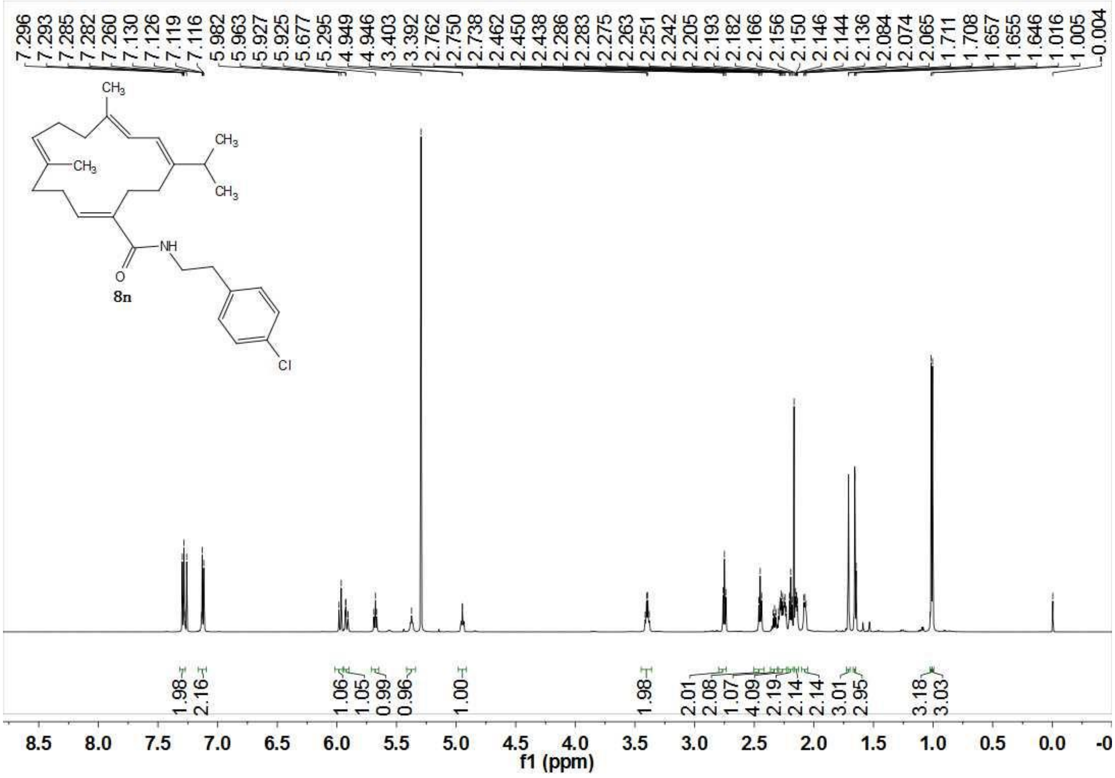

Figure S27. ^{1}H-NMR Spectrum for **8n** ($CDCl_3$, 600 MHz).

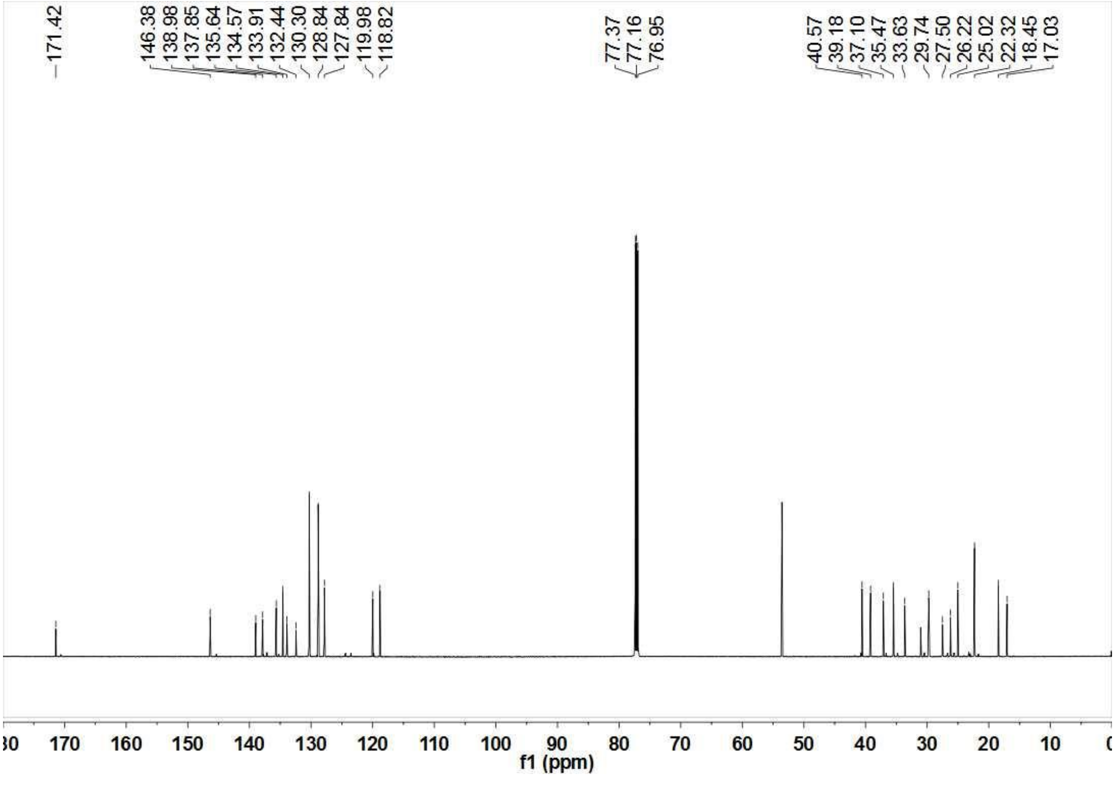

Figure S28. ^{13}C-NMR Spectrum for **8n** ($CDCl_3$, 150 MHz).

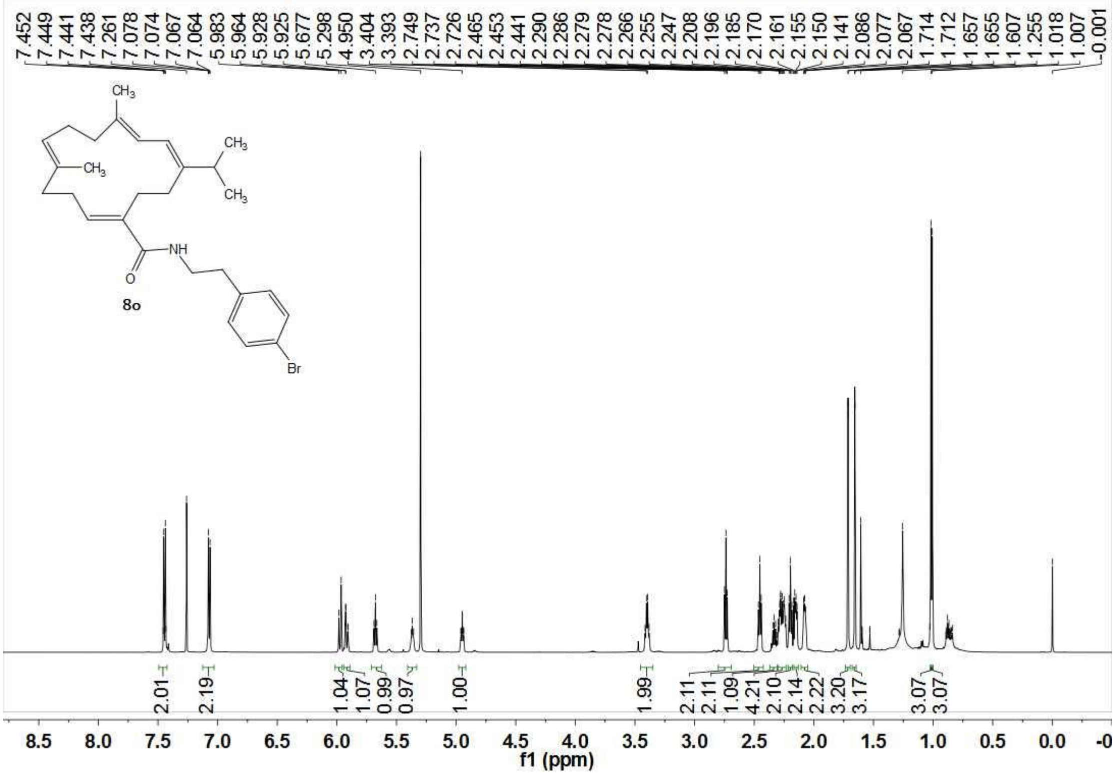

Figure S29. ^{1}H-NMR Spectrum for **8o** ($CDCl_3$, 600 MHz).

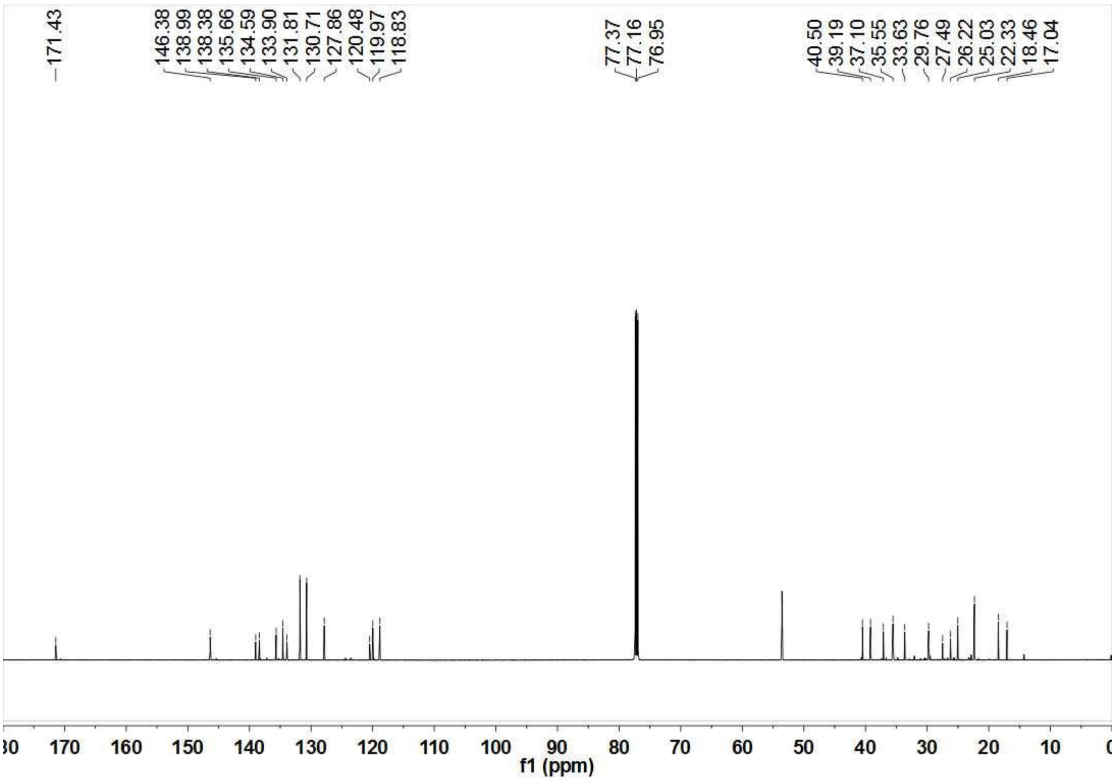

Figure S30. ^{13}C-NMR Spectrum for **8o** ($CDCl_3$, 150 MHz).

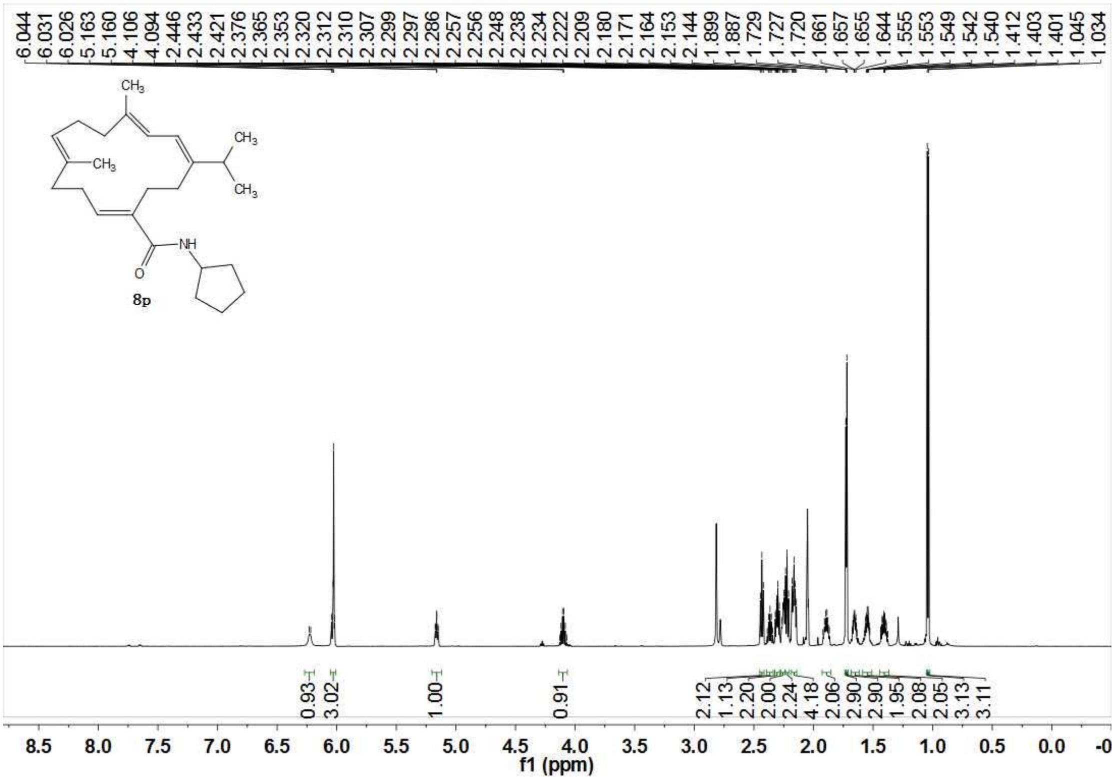

Figure S31. ^{1}H-NMR Spectrum for **8p** (CD_3COCD_3, 600 MHz).

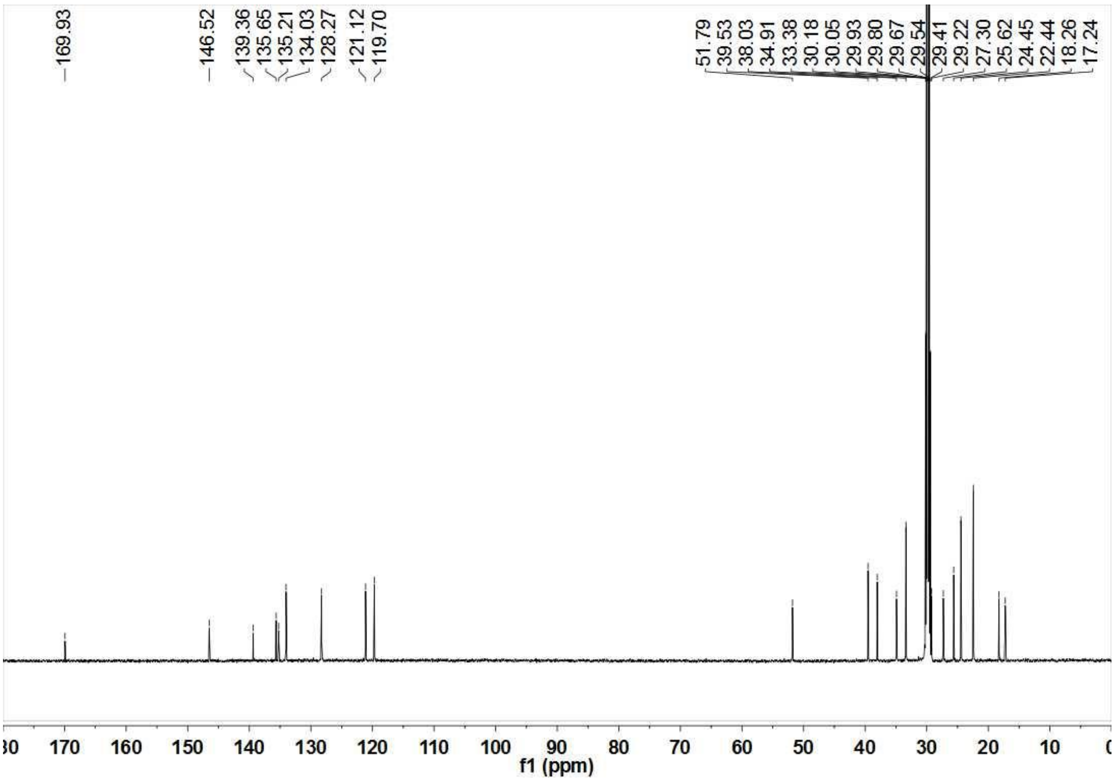

Figure S32. ^{13}C-NMR Spectrum for **8p** (CD_3COCD_3, 150 MHz).

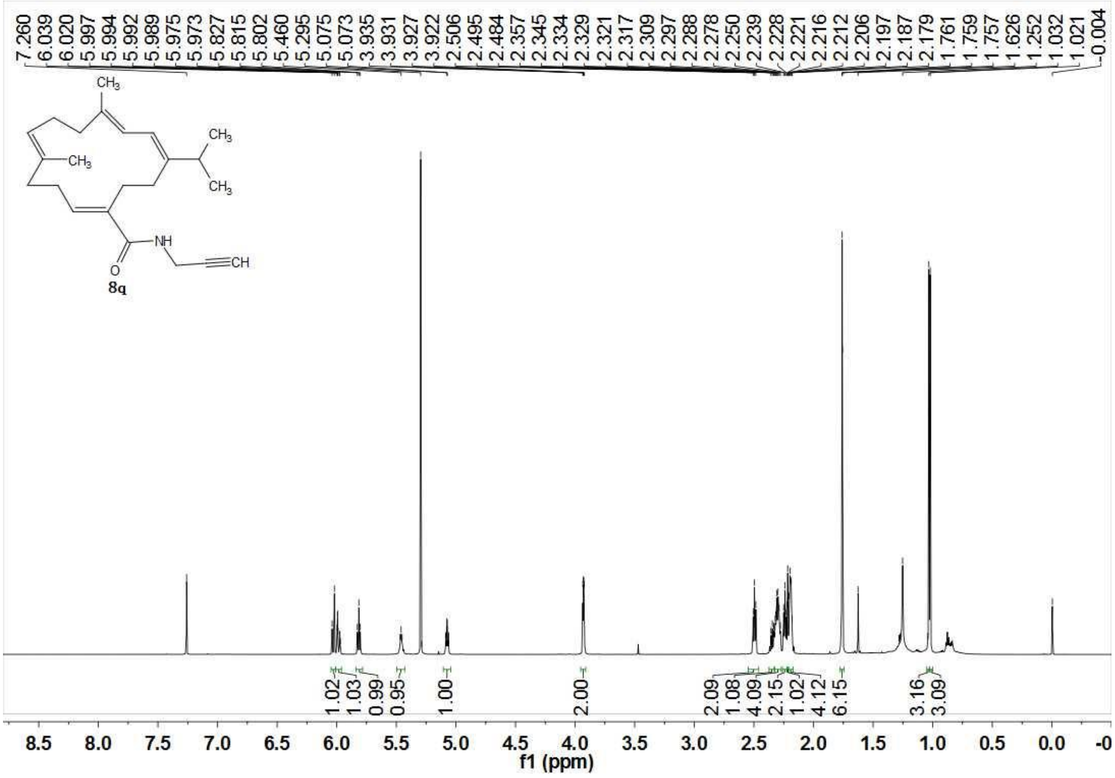

Figure S33. ^{1}H-NMR Spectrum for **8q** ($CDCl_3$, 600 MHz).

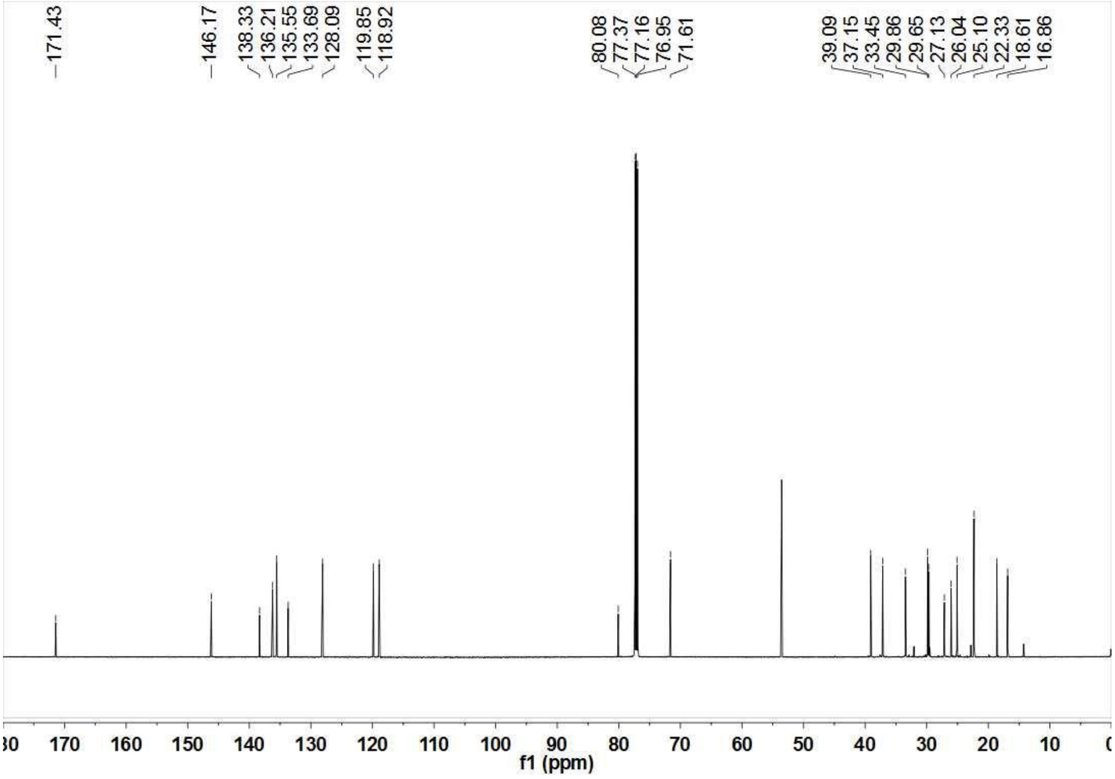

Figure S34. ^{13}C-NMR Spectrum for **8q** ($CDCl_3$, 150 MHz).

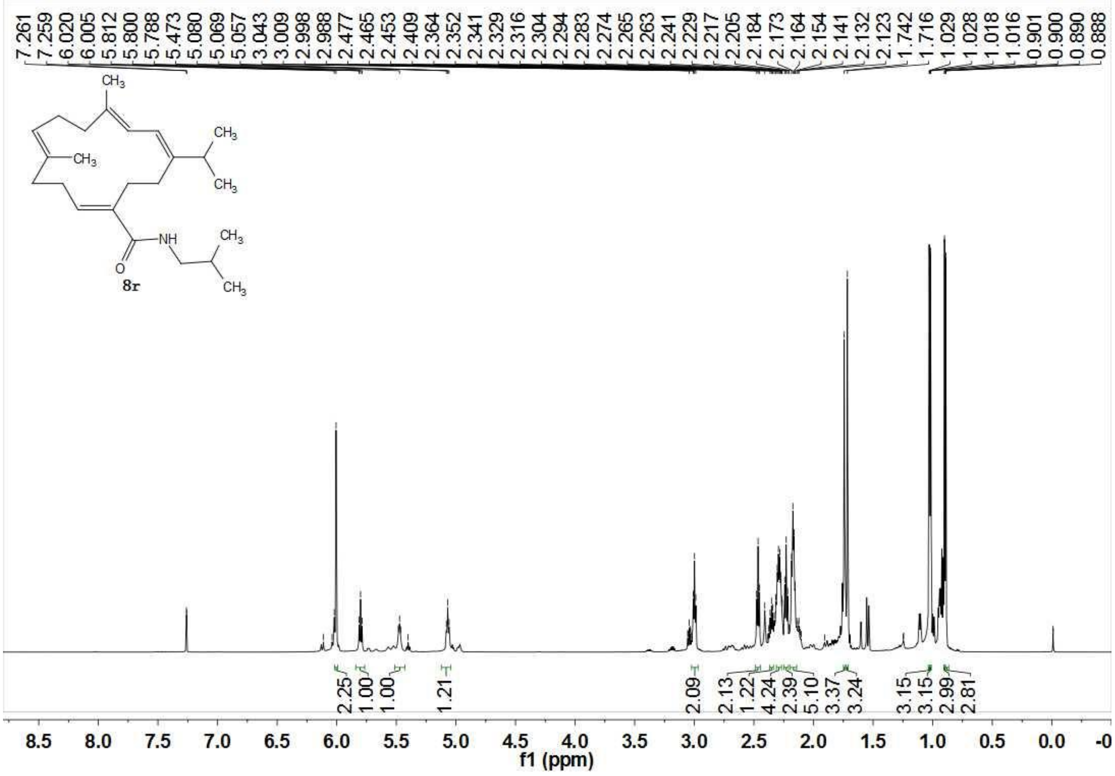

Figure S35. ^{1}H-NMR Spectrum for **8r** ($CDCl_3$, 600 MHz).

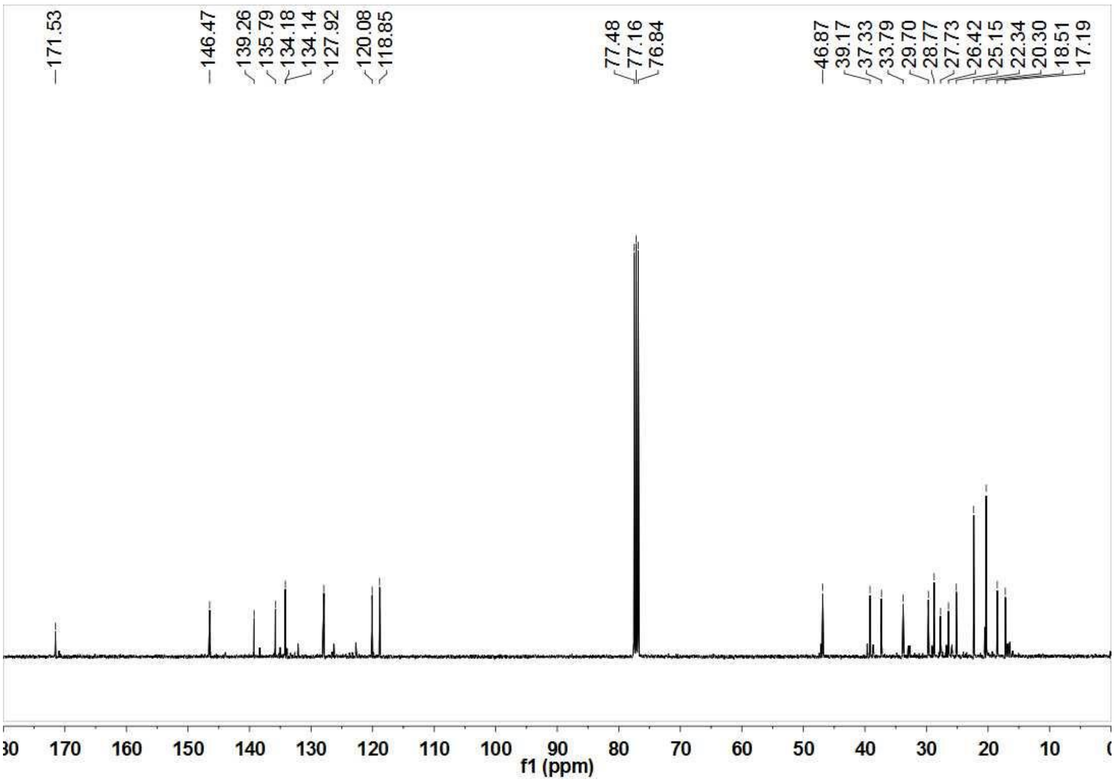

Figure S36. ^{13}C-NMR Spectrum for **8r** ($CDCl_3$, 100 MHz).
